# Unraveling the influence of trial-based motivational changes on performance monitoring stages in a flanker task

**DOI:** 10.1038/s41598-023-45526-0

**Published:** 2023-11-06

**Authors:** Rebecca Overmeyer, Hans Kirschner, Adrian G. Fischer, Tanja Endrass

**Affiliations:** 1 Chair for Addiction Research, Faculty of Psychology, Institute of Clinical Psychology and Psychotherapy, Technische Universität Dresden, Chemnitzer Straße 46a, 01187 Dresden, Germany; 2 Institute of Psychology, Otto-von-Guericke University, Magdeburg, Germany; 3 Department of Education and Psychology, Freie Universität Berlin, Berlin, Germany; 4 Neuroimaging Center, Technische Universität Dresden, Dresden, Germany

**Keywords:** Psychology, Human behaviour, Electrophysiology, Motivation, Reward, Cognitive neuroscience, Attention, Cognitive control, Motivation

## Abstract

Performance monitoring (PM) is a vital component of adaptive behavior and known to be influenced by motivation. We examined effects of potential gain (PG) and loss avoidance (LA) on neural correlates of PM at different processing stages, using a task with trial-based changes in these motivational contexts. Findings suggest more attention is allocated to the PG context, with higher amplitudes for respective correlates of stimulus and feedback processing. The PG context favored rapid responses, while the LA context emphasized accurate responses. Lower response thresholds in the PG context after correct responses derived from a drift–diffusion model also indicate a more approach-oriented response style in the PG context. This cognitive shift is mirrored in neural correlates: negative feedback in the PG context elicited a higher feedback-related negativity (FRN) and higher theta power, whereas positive feedback in the LA context elicited higher P3a and P3b amplitudes, as well as higher theta power. There was no effect of motivational context on response-locked brain activity. Given the similar frequency of negative feedback in both contexts, the elevated FRN and theta power in PG trials cannot be attributed to variations in reward prediction error. The observed variations in the FRN indicate that the effect of outcome valence is modulated by motivational salience.

## Introduction

Performance monitoring (PM) is the cognitive process of identifying situations where outcomes deviate from intended goals. The purpose of PM is to implement adaptations that compensate current problems and optimize actions in similar situations in the future^1,2^. PM enables flexibility and adjustments of behavioral strategies according to changing contextual demands. For example, we might be extra careful in a situation where there is much to lose, and behave riskier in situations that offer significant incentives but do not pose any danger. This study examines whether and how PM-related brain activity is affected by changes in motivational context, specifically potential gain and loss avoidance.

The effects of changes in motivational context on behavior have been studied with various task designs that manipulate rewards and feedback^3–6^. These manipulations can include block designs as well as trial-by-trial changes in motivational context. Incentive prospect has been associated with better task performance and may be modulated by task-relevant attentional processes^6–9^. Reward incentives appear to be most effective in regulating and enhancing cognitive control performance in a proactive and preparatory manner^10,11^. However, rewards also seem to improve response inhibition without preparatory cues and proactive mechanisms being involved^12^. Sokol-Hessner and Rutledge^13^ formulated a model that links dopamine to risk taking for rewards and norepinephrine to loss aversion in punishment. The model predicts that behavior in gain vs. loss contexts is differentially sensitive to manipulations of the dopaminergic and noradrenergic system. The expected value of control (EVC) theory^14^ suggests that individuals adjust the allocation of control to maximize their expected reward and minimize their expected costs for exerting control. Thus, the adjustment of control strongly depends on motivational factors and therefore it is critical to understand the effects of motivational context on behavior and control allocation^15^. Rewarding or gain context may invigorate approach behavior, whereas punishment or loss context lead to cautious behavior and avoidance. This has been shown experimentally, where higher rewards resulted in faster responses and higher punishments in higher accuracy^16^. Contextual factors moderate these mixed or bundled incentive effects, and clear representations of the motivational context aid in determining the control allocation strategy and amount of effort needed to achieve the goal—the same outcome may strengthen or weaken responses, depending on motivational valence and context^11,15^. Moreover, drift–diffusion models (DDM) provide a useful framework for assessing the influence of different types of incentives (e.g., reward, punishment) on distinct adjustments in control allocation^17,18^. Examples of this would be higher evidence accumulation rate and lower response threshold for rewarding trials, indicating faster information processing to potentially maximize response rate, and a higher threshold for punishment trials, indicating response caution^16^. Interestingly, it has been shown that only trial-based changes of motivational context affected performance, as indicated by higher accuracy, in an arrow flanker task^19^, which is why we manipulated motivational context in a trial-wise fashion in the current study.

In the scalp-recorded event-related potentials, a quite uniform sequence of negative and positive deflections has been found for different situations when adaptation is needed ranging from stimulus, over response to feedback processing^2^. An early frontocentral sequence of a negative followed by a positive ERP deflection is assumed to be generated at least in part in the posterior medial frontal cortex and associated with a fast alarm signal for the potential necessity for adaptation. This is followed by a more sustained parietal positivity presumably reflecting more elaborated processing of the evaluation signal^2^. Neural correlates of PM have been shown to be sensitive to motivational context. For stimulus processing, higher N2 amplitudes have been reported in punishment compared to reward motivated trials in a flanker task with trial-by-trial reward manipulation^20^. Within the punishment context, the P3 also exhibited higher amplitudes for congruent trials than for incongruent trials^20^. Another study using a Stroop-like task did not observe differences in P3 between trial-by-trial changing contexts^21^. Regarding response processing, more significant errors, and also higher reward incentives, have been found to be associated with enhancements in error-related negativity (ERN) amplitude—the ERN was therefore suggested to be sensitive to motivation^22^. Similar results have been found for manipulations of punishment: ERN amplitudes increased in trials with a threat of punishment, compared to neutral trials^23,24^. When comparing different motivational contexts, results are mixed: in one study using a flanker task with trial-by-trial reward manipulations with the stimulus itself indicating the context, ERN amplitude was reported to be higher in trials with monetary loss than in trials with monetary gain omission^20^. In another study utilizing a block design in a Simon task, the opposite was reported: the ERN amplitude was higher in the reward block for monetary gain omission than in the punishment block for monetary loss^25^. Other studies found no difference between reward and punishment contexts for the ERN^26–28^. Aside from the ERN, results on the Pe are also mixed: there are reports of the Pe being larger in reward trials compared to punishment trials, but also reports of no significant differences between Pe in different incentive contexts^20,26^. The Pe has also been reported to be larger in high punishment contexts compared to low punishment and neutral contexts^27^. Additionally, there is no evidence of an effect of motivational context on the correct-related negativity [CRN;^20,29^], a smaller negativity that is observed following correct response execution^22,30,31^.

Feedback processing is particularly sensitive to motivational context. Here, reward prediction errors (RPE), feedback valence and surprise are likely multiplexed in the FRN^32–40^. The FRN has additionally been found to differ depending on the context of the outcome, with the FRN appearing to correspond to the relative value of the outcome when compared to other potential outcomes, as well as motivational salience^40–42^. Studies suggest that FRN modulations do not depend on whether positive and negative feedback involves gain vs. omitted gain (gain context) or avoided vs. actual loss [punishment context;^20,21,25,41,43^, but see^44^]. Interestingly, there have been studies examining the differential influences of feedback magnitudes or valence and expectancy. The FRN was found to be determined by valence as well as expectancy, and to be influenced by valence and magnitude ambiguity^38,45,46^. FRN amplitudes did not differ between contexts when predictive accuracy was emphasized, but when outcome-valence was highlighted, worse-than-expected outcomes elicited higher amplitudes than better-than-expected outcomes^47^. This could also explain the conflicting results, aside from different tasks and reward manipulations. The feedback-related P3 also appears to be influenced by gain and loss. The P3 has also been found to be sensitive to magnitude and validity of feedback^46,48,49^. When contrasting gain and loss contexts, both no differences between contexts and higher amplitudes for actual gain or loss have been reported^20,21,43^. For comparisons of P3 amplitude in loss, loss avoidance, gain and gain omission, presence or direction of effects also vary between studies^20,21,43^.

An alternative explanation for the difference in feedback ERPs suggests that the difference reflects variations in reward processing and terms the difference between positive and negative feedback as the reward-related positivity (RewP)^50^. The RewP has been proposed to reflect a positive RPE signal^32,33,51^. The RewP has also been linked to motivational factors concerning task performance. It has additionally been shown to be sensitive to differences in reward values associated with different motivational contexts in a gambling task^33,52^. In recent years, the discussion has also shifted towards integrating ERP analyses with accounts of time–frequency associations and distinguishing between activity for gains and activity for losses, with the ERP measure of the RewP being predicted independently by loss-related theta power and gain-related delta power, with both possibly contributing differently to the ERPs^53–56^. Similarly, it has been suggested that the ERPs after feedback result from two overlapping non-valenced RPE magnitude responses: a frontal theta FRN on losses and a posterior delta RewP on wins^57^. Additionally, frontal midline theta power has been associated with both cognitive effort and the exertion of cognitive control, fitting well into the framework of PM^58,59^.

**Figure 1:**
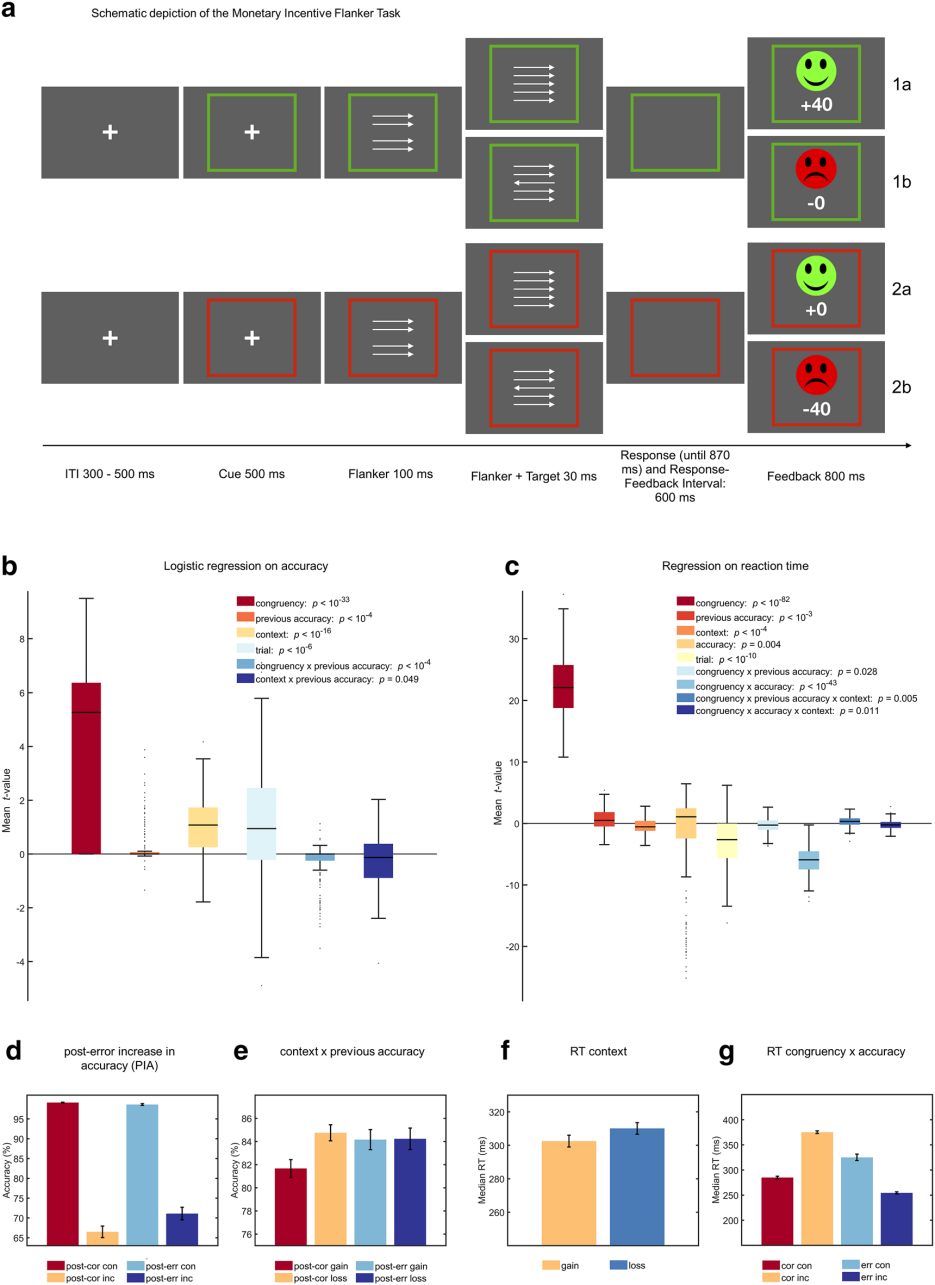
(**a**) Schematic depiction of the Monetary Incentive Flanker Task. Participants were instructed to respond with the left or right button according to the direction of the middle arrow. In the potential gain (PG) context, the 80% fastest correct responses were rewarded (1a), in the loss avoidance context, the 80% fastest correct responses were not punished (2a). Incorrect and the 20% slowest correct responses were either not rewarded (1b), or punished (2b). Logistic regression on accuracy (**b**) and multiple single-trial regressions on RT (**c**) were used to assess behavioral effects in the task, controlling for confounding variables and the interdependence of effects. Interference effects are reduced after errors with regard to accuracy (**d**). Motivational context predicted RT, in that the loss context increased accuracy, as participants only showed post-error increase in accuracy (PIA) within the PG context (**e**), as well as RT (**f**). Stimulus incongruence decreased accuracy and increased RT, and interacted with accuracy (**g**). **b**, **c** depict mean within participant* t*-values, *p*-values are derived from t-tests of individual regression weights against zero and were Bonferroni corrected. RT is calculated as the mean of within-participants median RTs per condition. For **b**, **c**, boxes = interquartile range (IQR),  −  = median, whiskers = 1.5 × IQR, gray dots = outlier. For **d–g**, error bars represent the standard error of the mean. See Supplementary Table 1 for descriptive statistics of behavioral data.

The aim of the present study was to examine whether and how PM-related brain activity is influenced by motivational context. Critically, we investigated this with trial-based potential incentives in a potential gain (PG) context and a loss avoidance (LA) context, using electroencephalography (EEG) in a monetary incentive version of the flanker task [see Figure 1;^60,61^]. We were interested in PM at different stages: stimulus-related (N2, stimulus-P3), response-related (ERN, early and late Pe), and feedback-related activity (FRN, P3a, P3b). We conducted time–frequency analyses for feedback processing, to explore possibly different motivational effects on theta and delta activity. In addition, we examined behavioral effects and used a drift–diffusion model (DDM) to assess the influence of motivational context (e.g., reward, punishment) on distinct adjustments of behavioral control allocation^17,18^. These analyses were carried out on a dataset that has been analyzed before, with a different focus of analysis [see^62^]. As the findings on ERPs reported in the literature are very heterogeneous, and as utilized tasks differ substantially, our analyses regarding context effects were exploratory.

## Results

### Behavioral effects

To assess participants behavior in the task (for a schematic depiction of the task see Fig. 1a) and to examine post-error adjustments like post-error slowing (PES), post-error increase in accuracy (PIA), and post-error speeding for incongruent trials (PERI; post-error reduction of interference)^63,64^, we conducted two multiple robust single-trial regressions. Errors were defined as actual false responses. We regressed critical task factors onto participant’s single-trial accuracy (GLM1) and reaction time (RT; GLM2) while controlling for confounds and the interdependence of effects, similar to Fischer et al.^65^. Individual regression weights were then compared using two-sided *t*-tests corrected for multiple comparisons on group level.

Findings were representative of flanker tasks and additionally reflected effects of motivational context (Fig. 1). Effects of interference were reflected in slower (*t*_129_ = 47.93, *p* < 10^–82^) and more erroneous (*t*_129_ = 17.08, *p* < 10^–33^) responses on incongruent trials. PES was reflected in a significant main effect for *previous accuracy* (*t*_129_ = 4.27, *p* < 10^–3^). Moreover, there was a general slowing in the LA compared to the PG context (*t*_129_ = − 4.92, *p* < 10^–6^). Additionally, PIA was present for the PG, but not for the LA context (Fig. 1d, interaction *context x previous accuracy t*_129_ = − 2.68, *p* = 0.049). Errors on the previous trial led to increased accuracy on incongruent, but not congruent trials (Fig. 1, interaction *congruency x previous accuracy t*_129_ = − 4.94, *p* < 10^–4^).

In a post-task questionnaire, participants also reported that they prioritized accuracy (*M* = 81.13, *SD* = 18.30) over speed (*M* = 73.38, *SD* = 20.02), *z* = 4.71, *p* < 0.001, *rc* = 0.49, on visual analogue scales. They also reported paying more attention to the green square cueing the PG context (*M* = 47.38%, *SD* = 31.22), compared to the red square cueing the LA context (*M* = 40.16%, *SD* = 29.38), *z* = − 3.45, *p* < 0.01, *rc* = 0.39. For descriptive RT analyses (see Supplementary Table 1), trials were categorized into post-correct (correct trials following correct trials), pre-error (correct trials before error commission), error, and post-error (correct trials after erroneous response) trials [see^66^].

### Drift–diffusion modelling

**Figure 2:**
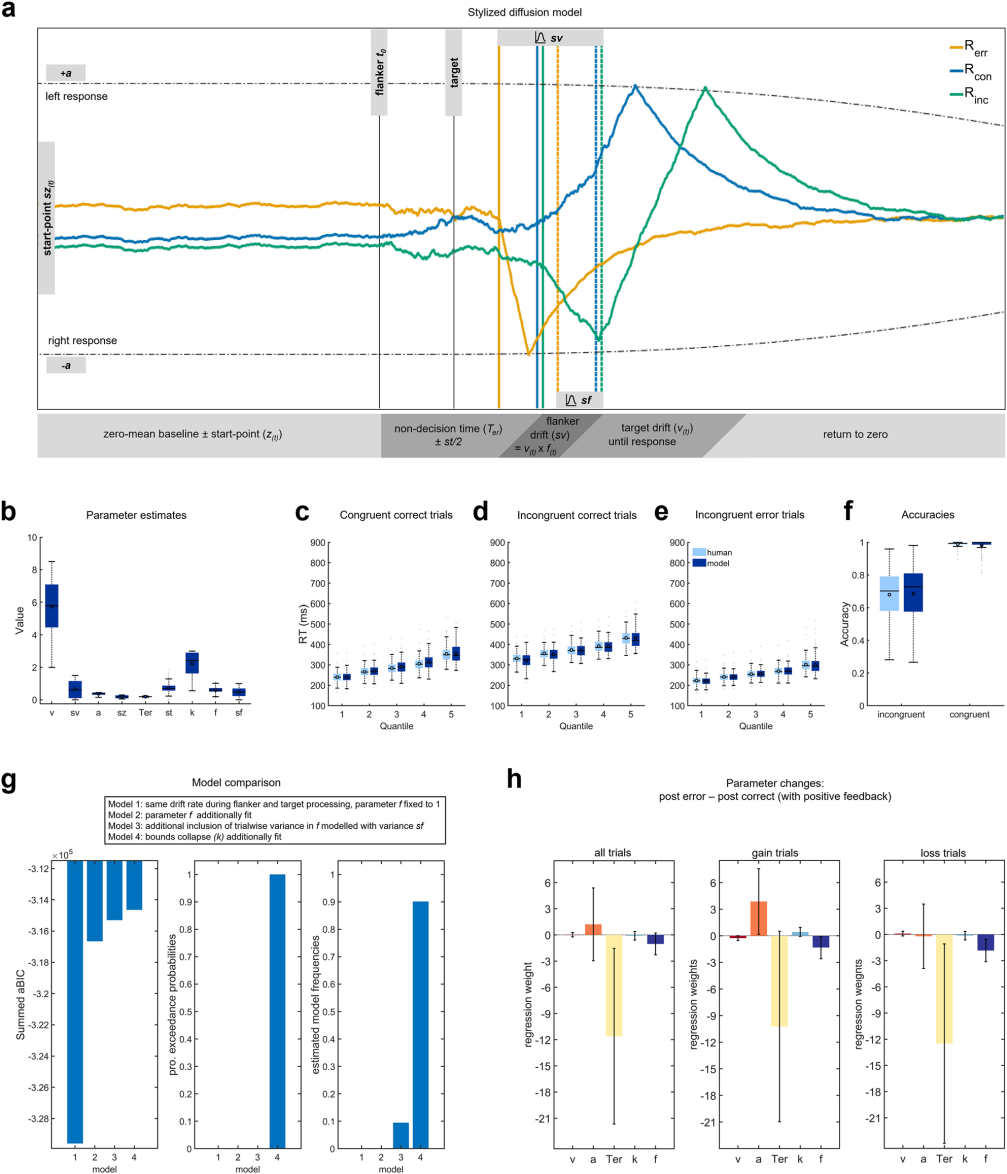
(**a**) Stylized illustration of the winning multi-stage drift diffusion model (DDM) based on simulated data. Depicted are three decision processes: incongruent correct trials (R_inc_, green decision process), congruent correct trials (R_con_, blue decision process), and incongruent error trials (R_err_, yellow decision process). The DDM consisted of five separate stages (depicted as gray bars underneath) and included nine free parameters: drift rate (*v*), variance in drift rates (*sv*), boundary (*a*), variance in start points (*sz*), non-decision time (*Ter,* reflecting visual processing and motor execution times), variance in *Ter* (*st*), bounds collapse (*k*), flanker weighting (*f*), and variance in *f* (*sf*). *Stage 1* represents a pre-stimulus baseline. On each trial the decision process starts with a random start point *sz*, which is drawn from a uniform distribution. *Stage 2* represents the non-decision time (*Ter)* per trial. *Ter* varies on each trial depending on *st*, which is also modelled as a uniform distribution. In this period the decision process randomly drifts away from the start point. *Stage 3* reflects a noisy diffusion in flanker direction (sv), which varies from trial-to-trial (*sf)*. Both are modelled as Gaussian distributions. Thus, on each trial in this stage, the decision process drifts in the flanker direction with drift rate *v*_*(t)*_ x *f*_*(t)*_. *Stage 4* represents the diffusion into the correct direction (*v*_*(t)*_). Finally, *stage 5* reflects the return to the baseline of the decision variable. For more details on the utilized DDM, see Fischer et al.^65^ and Kirschner et al.^67^. This model makes specific predictions: longer RTs for incongruent correct trials are due to an initial drift away from the correct response (green decision process). Error likelihood increases when *f(t*) is higher on a given trial resulting in an early crossing of decision boundaries (yellow decision process). As a result, errors are usually faster on incongruent trials. The height of the boundary parameter (*a*) determines how much evidence accumulation is required to cross the boundary and trigger a response and collapses with increasing decision time (scaled by *k*). To simulate the temporal evolution of the single trial decision process depicted we used the mean maximum likelihood parameters from the group-fit obtained for the dynamic bound model (DDM4). *Note*: To speed up the model fitting procedure, we did neither simulate baseline periods nor return to baseline during the fitting because these have no effect on model predictions. **b-g** DDM parameters and fit. **b** depicts DDM parameter values for the winning model including nine free parameters. **c-e** Quantile fits of the winning model (dark blue) against human reaction time data (light blue). **f** Model and human accuracy. **g** Model comparisons. Cumulated approximated Bayesian Information Criterion (aBIC) scores over participants of each candidate model. Higher values indicate a better fit of the models to the behavioral data. Results indicate that the dynamic bound model (DDM4) provides the best fit to the data. Protected exceedance probabilities (pEP) similarly favor DDM4. **h** Individual contributions of model parameter changes were compared using logistic regression on trial type (depicted here post error trials vs. post correct trials that received positive feedback for all trials, potential gain trials and loss avoidance trials). Plotted are regression weights and error bars reflect 95% CI.

In an exploratory analysis, we aimed to parse out the decision process that gives rise to the context dependent behavioral adaptations, by fitting a nested set of different multistage DDMs to task behavior. DDMs provide a framework to investigate distinct adjustments of behavioral control allocation. The details of these models have been describe previously^65,67^ and are shown in Fig. 2a. The winning model (as measured by the approximated BIC and achieving protected exceedance probabilities (pEP) of 1, see Fig. 2g) was one that included all features (see Fig. 2a for illustration of model features; see Fig. 2b for parameter estimates for the winning model). Sufficiency of the model was evaluated through post predictive checks that matched participants RT and accuracies on various task factors (see Fig. 2c–f). Moreover, parameter recovery analyses revealed that our fit procedure reliably recovered the parameters that generated synthetic data (see Supplementary Fig. 1). Next, we fit the winning model separately to post-error and post-correct trials with positive and negative feedback, respectively, across all contexts and within the PG and LA context. Here, we fixed the variance parameters (*sv, sz, st, sf*) to the group mean to facilitate convergence. These models captured context-specific RT and accuracies well (see Supplementary Fig. 2). We then used a set of multiple logistic regression of parameter values (drift rate (*v*), boundary (*a*), non-decision time (*T*_*er*_), flanker suppression (*f*), boundary collapse (*k*)) onto the trials the model was fitted to, to investigate context related adaptations. Positive regression coefficients indicate parameter increases following errors. We found that *T*_*er*_ was decreased after errors compared to correct responses with positive feedback (*β* = − 11.61, *SE* = 5.11, *OR* = 0.01), *T*_*er*_ was also decreased after errors for the LA context (*β* = − 12.50, *SE* = 5.80, *OR* = 0.01), as was flanker weighting (*β* = − 1.84, *SE* = 0.65, *OR* = 0.16). For PG trials, the same comparison exhibited similar parameter changes for *f* (*β* = − 1.32, *SE* = 0.64, *OR* = 0.27), and an additional increase in *a* (*β* = 3.87, *SE* = 1.88, *OR* = 48.02). See Fig. 2h for a visualization. Also, *k* was decreased after correct trials with negative feedback compared to correct trials with positive feedback (*β* = 0.50, *SE* = 0.24, *OR* = 1.64), which fits with negative feedback in this case signaling responding that was too slow. All other trial types did not differ in fitted parameters. Taken together, context specific adjustments to the decision process were only observed for the boundary *(a)* which is increased after errors in the PG context. This fits with our other behavioral results showing lower accuracy in general as well as in trials after correct responses in the PG context but no change in accuracy in the LA context, indicating a more cautious response style.

### EEG analysis

We employed single-trial robust regression to obtain a regression weight time-course for PM-related EEG activity for all electrodes^68^. See Table 1 for a summary of regression effects by motivational context.

**Table 1:** Summary of context effects.

				Test statistic	*p*
Stimulus processing					
P2	PG	>	LA	4.03	8.9979e^−5^
N2	PG	≅	LA	1.49	n.s
P3a	PG	>	LA	7.13	6.8248e^−11^
P3b	PG	≅	LA	− 1.97	n.s
Response processing					
ERN	PG	≅	LA	3.46	n.s
Early Pe	PG	≅	LA	− 2.57	n.s
Late Pe	PG	≅	LA	3.47	n.s
Feedback processing					
Negative feedback					
P2	PG	>	LA	6.50	1.5757e^−9^
FRN	PG	>	LA	− 7.62	4.8062e^−12^
P3a	PG	>	LA	12.31	1.8517e^−23^
P3b	PG	≅	LA	− 2.42	n.s
Theta (FCz)	PG	>	LA	13.53	1.6092e^−26^
Theta (Pz)	PG	≅	LA	3.50	n.s
Delta (FCz)	PG	≅	LA	6.93	n.s
Delta (Pz)	PG	≅	LA	− 3.15	n.s
Positive feedback					
P2	PG	>	LA	6.12	1.0570e^−8^
FRN	PG	≅	LA	− 0.78	n.s
P3a	PG	<	LA	− 3.92	1.4553e^−4^
P3b	PG	<	LA	− 7.81	1.7257e^−12^
Theta (FCz)	PG	<	LA	− 7.47	1.0978e^−11^
Theta (Pz)	PG	<	LA	− 4.68	7.2528e^−6^
Delta (FCz)	PG	>	LA	3.99	1.0880e^−4^
Delta (Pz)	PG	≅	LA	2.00	n.s

PG, potential gain context; LA, loss avoidance context; ERN, error-related negativity; early Pe, early error positivity; late Pe, late error positivity; FRN, feedback-related negativity; Theta, theta power; Delta, delta power; > , effect for potential gain context is higher; < , effect for loss avoidance context is higher; ≅ , no significant effect; test statistic, largest t value (*t*) for effect; *p*, corresponding *p* value; n.s., non-significant.

#### Stimulus-locked

**Figure 3:**
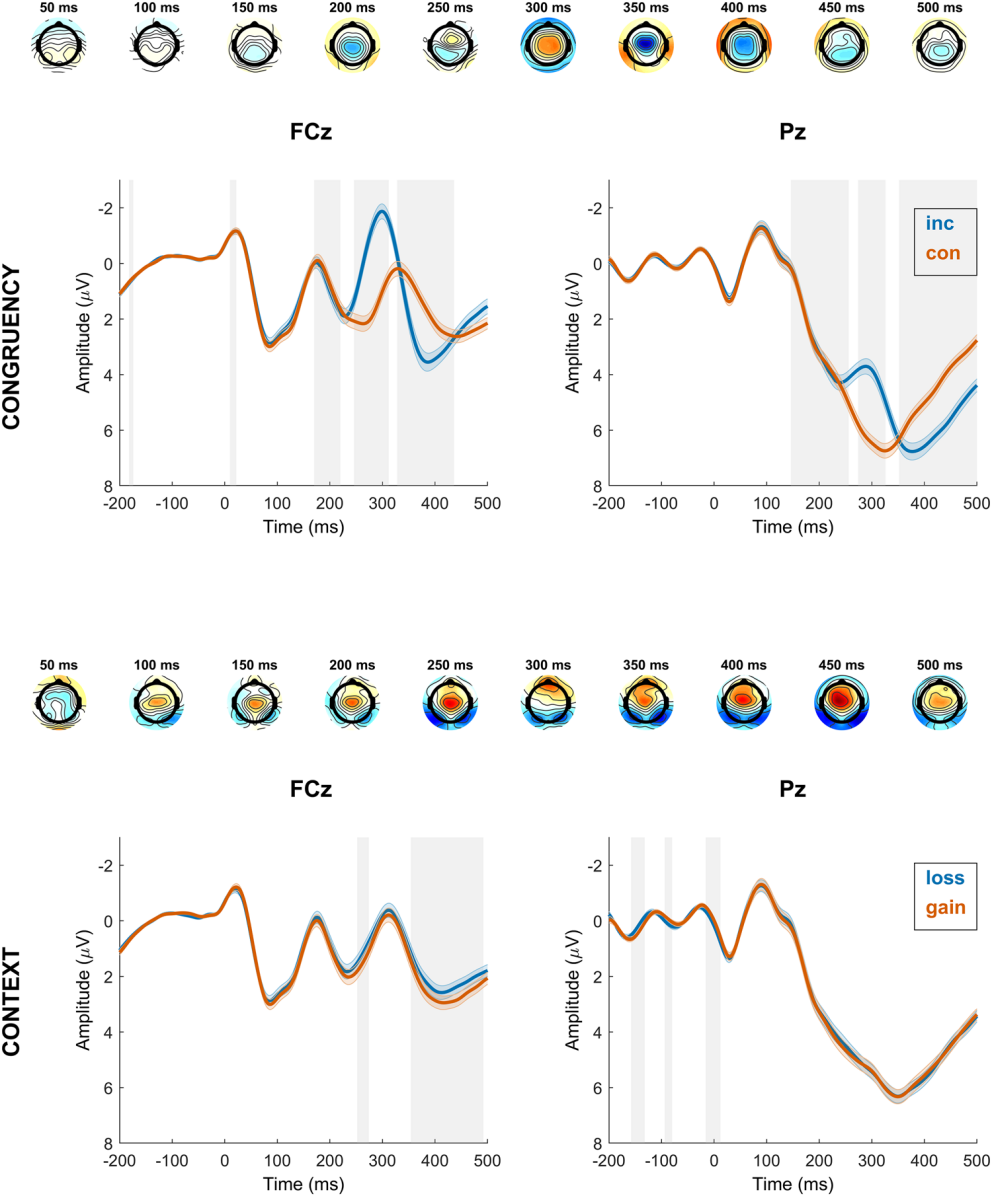
Time course of stimulus-locked regression effects for significant regressors congruency (incongruent, congruent) and context (loss, gain). The first and third row represent topographical maps of the associations between EEG activity and the respective regressor (first row: congruency; third row: motivational context). Grand average event-related potential (ERP) waveforms at electrodes FCz and Pz are depicted in the second (regressor: congruency; blue lines indicate incongruent trials, orange lines indicate congruent trials) and fourth row (regressor: context; blue lines indicate loss avoidance trials, orange lines indicate potential gain trials), stimulus-locked (at 0 ms) for correct trials. Shades indicate the SEM. Gray shading behind the waveforms indicates significance of regression weights at *p* < 2.2654e^−4^ (FCz) and *p* < 3.2189e^−4^ (Pz). Note that regular ERPs do not account for other task factors.

The stimulus-locked model (with congruency, context and their interaction as predictors within correct trials) revealed a significant effect of context at frontocentral electrodes in a time window corresponding to the P3a (354–492 ms) with the amplitude being more positive in the PG context. There also was a significant effect of context at frontocentral electrodes in the P2 time window (252–274 ms), with the amplitude also being more positive in the PG context^69,70^. The interaction between congruency and context was not significant. See Fig. 3 for a visualization of results.

#### Response-locked

**Figure 4:**
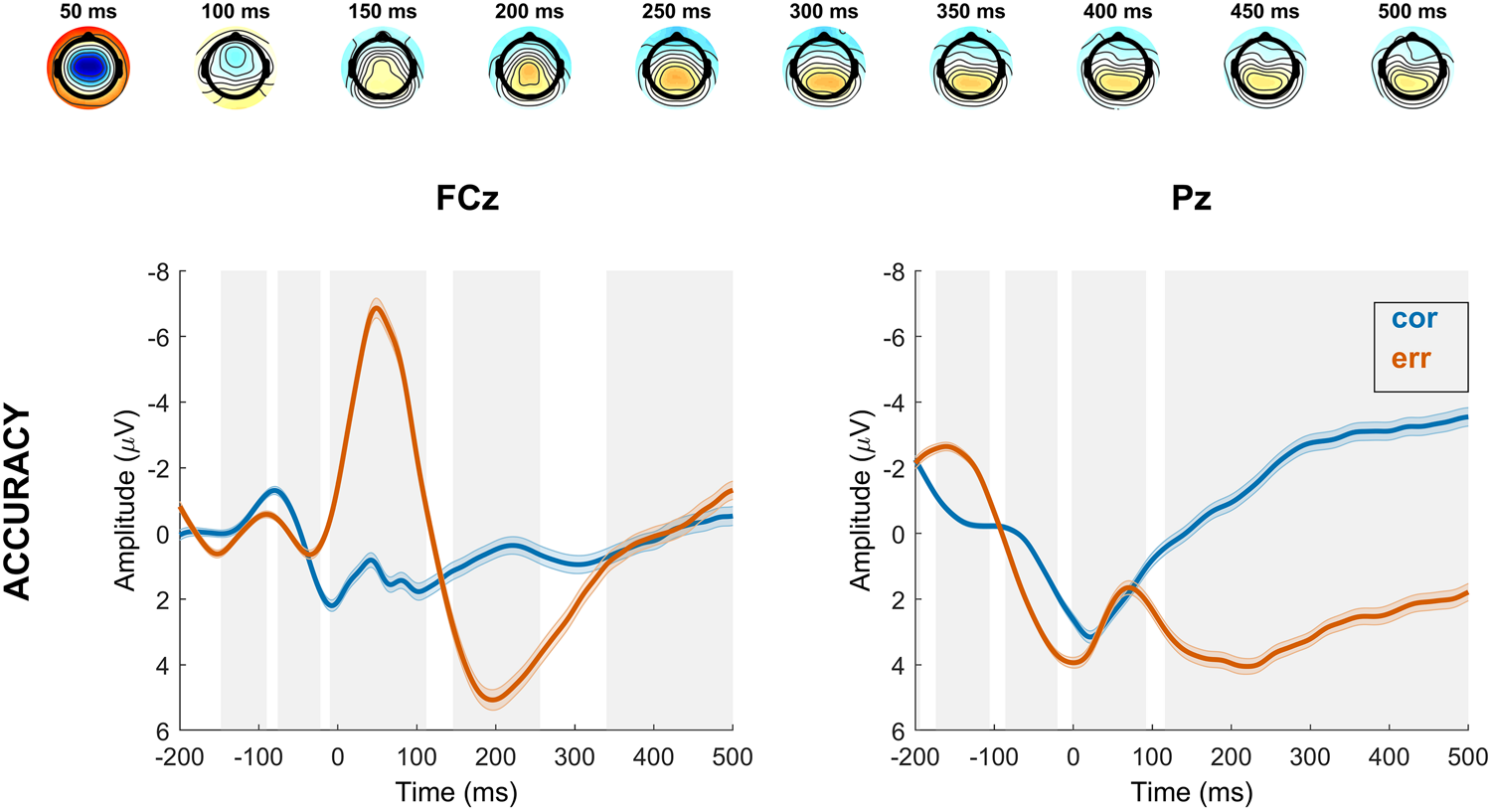
Time course of response-locked regression effects for significant regressor accuracy (correct, error). The first row represents topographical maps of the associations between EEG activity and the accuracy regressor. Grand average event-related potential (ERP) waveforms at electrodes FCz and Pz are depicted in the second row, response-locked for incongruent trials. Shades indicate the SEM. Gray shading behind the waveforms indicates significance of regression weights at *p* < 2.8827e^−4^ (FCz) and *p* < 3.4804e^−4^ (Pz). Note that regular ERPs do not account for other task factors.

The response-locked model (with accuracy, context and their interaction as predictors within incongruent trials) revealed a significant effect of response accuracy, but no covariation of motivational context at frontocentral or parietal electrodes, indicating that ERN, as well as early and late Pe were insensitive to motivational context. There was no significant interaction effect between accuracy and context. See Fig. 4 for a visualization of results.

#### Feedback-locked

Our analysis of feedback-locked EEG activity (with feedback, context and their interaction as predictors within correct trials) revealed significant effects of feedback type and motivational context at frontocentral electrodes. Notably, time windows corresponding to the P2 (160–210 ms), the FRN (244–286 ms), and the P3a (316–396 ms) were affected. Additionally, there was a significant effect of context at parietal electrodes within the P3b time window (420–548 ms). The interaction between context and feedback was significant at frontocentral electrodes within the FRN time window (218–286 ms) and the P3a time window (316–412 ms), and also at parietal sites within the P3b time window (370–388 ms). No interaction effect was found in the P2 time window.

We further conducted separate follow-up models for positive and negative feedback to investigate their individual contribution. Both models revealed a significant effect of context at frontocentral electrodes for the P2 time window (168–204 ms for negative feedback, and 166–246 ms for positive feedback), indicating larger amplitudes in the PG context. The FRN was significantly larger in the PG context for negative feedback (230–290 ms), but there was no significant effect of context on EEG activity at frontocentral electrodes in a time window corresponding to the FRN for positive feedback. More attention was paid to negative feedback in the PG context (larger P3a; 314–406 ms), whereas more attention was allocated to positive feedback in the LA context (larger P3a; 380–394 ms). The P3b was larger in the LA context for positive feedback (352–600 ms), there was no effect of context for negative feedback.

**Figure 5:**
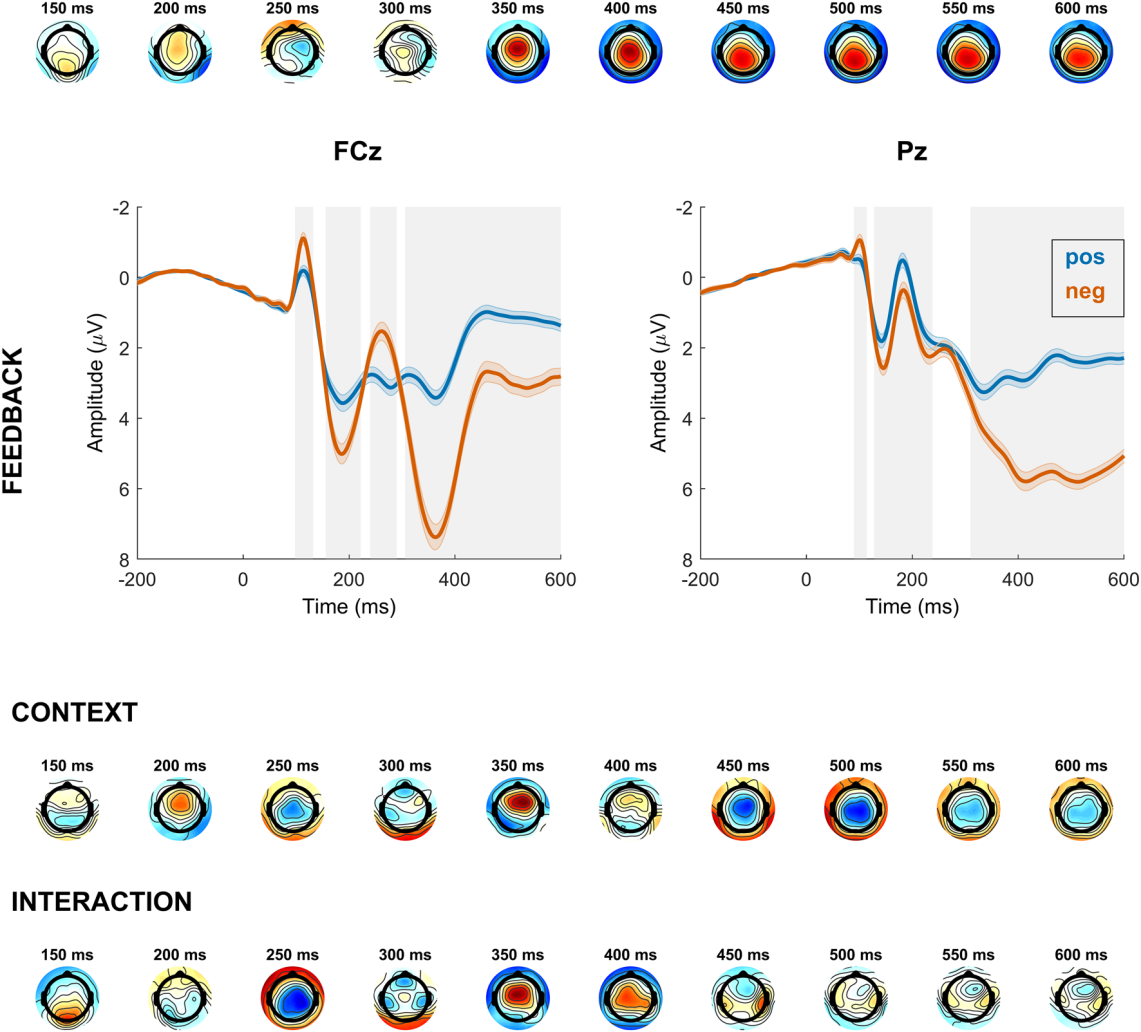
Time course of feedback-locked regression effects for significant regressors feedback (positive, negative), context (loss, gain) and the interaction of feedback and context. The first, third and fourth row represent topographical maps of the associations between EEG activity and the respective regressor. Grand average event-related potential (ERP) waveforms of the feedback regressor at electrodes FCz and Pz are depicted in the second row, feedback-locked for correct trials. Shades indicate the SEM. Gray shading behind the waveforms indicates significance of regression weights at *p* < 3.7708e^−4^ (FCz) and *p* < 2.7407e^−4^ (Pz). Note that regular ERPs do not account for other task factors.

**Figure 6:**
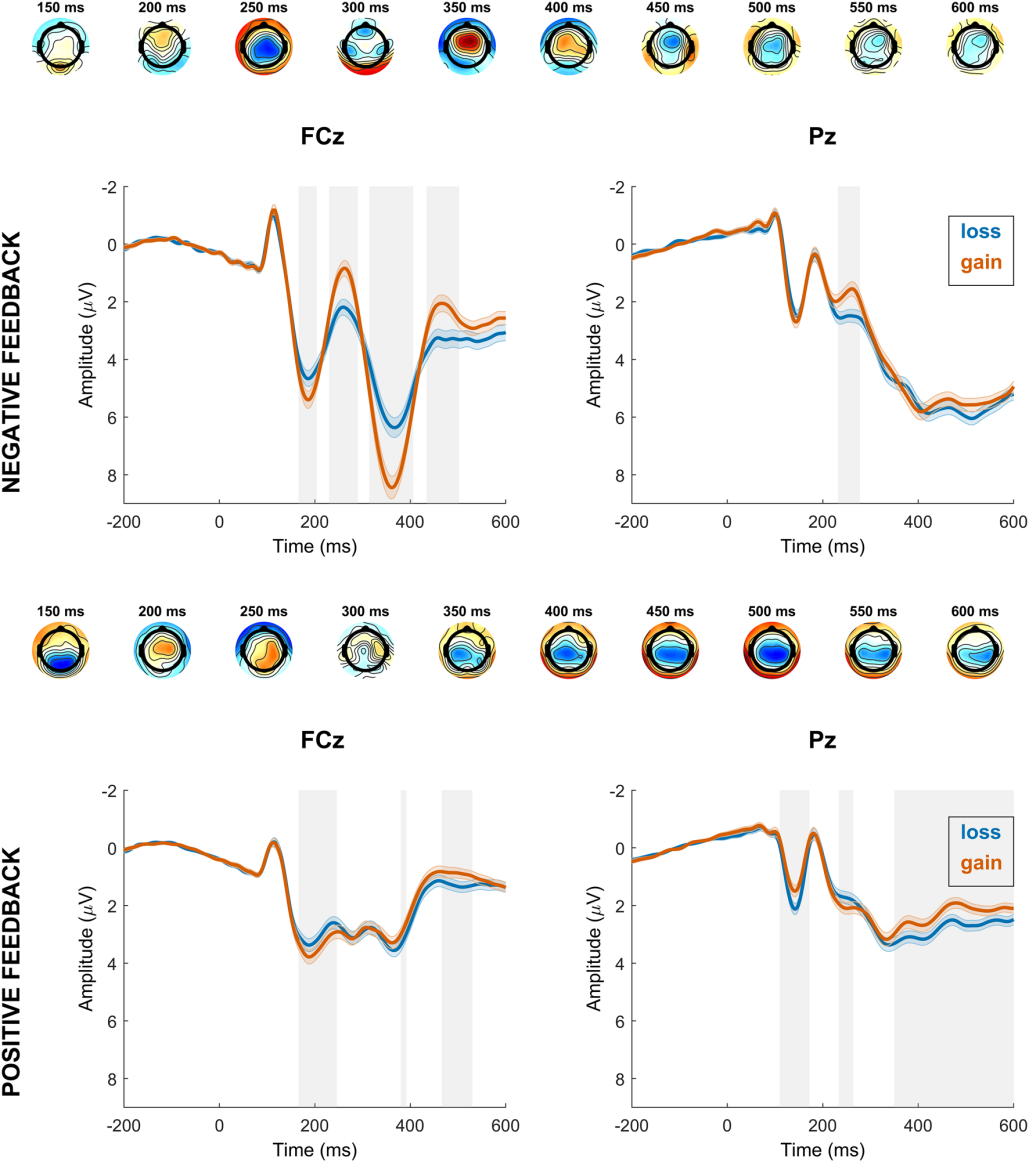
Time course of feedback-locked regression effects for significant regressor context (loss, gain) within correct trials with negative feedback and correct trials with positive feedback. The first and third row represent topographical maps of the associations between EEG activity and the context regressor within the respective trial type. Grand average event-related potential (ERP) waveforms at electrodes FCz and Pz are depicted in the second and fourth row, feedback-locked for correct trials with negative or positive feedback. Shades indicate the SEM. Gray shading behind the waveforms indicates significance of regression weights at *p* < 2.2664e^-4^ (FCz) and *p* < 2.1000e^-5^ (Pz) for negative feedback, and *p* < 1.9288e^-4^ (FCz) and *p* < 4.2192e^-4^ (Pz) for positive feedback. Note that regular ERPs do not account for other task factors.

For a visualization see Figs. 5 and 6. See the Supplement for an analysis of positive vs. negative feedback within each context, and an analysis of feedback after response errors.

#### Feedback-locked time–frequency analysis

**Figure 7:**
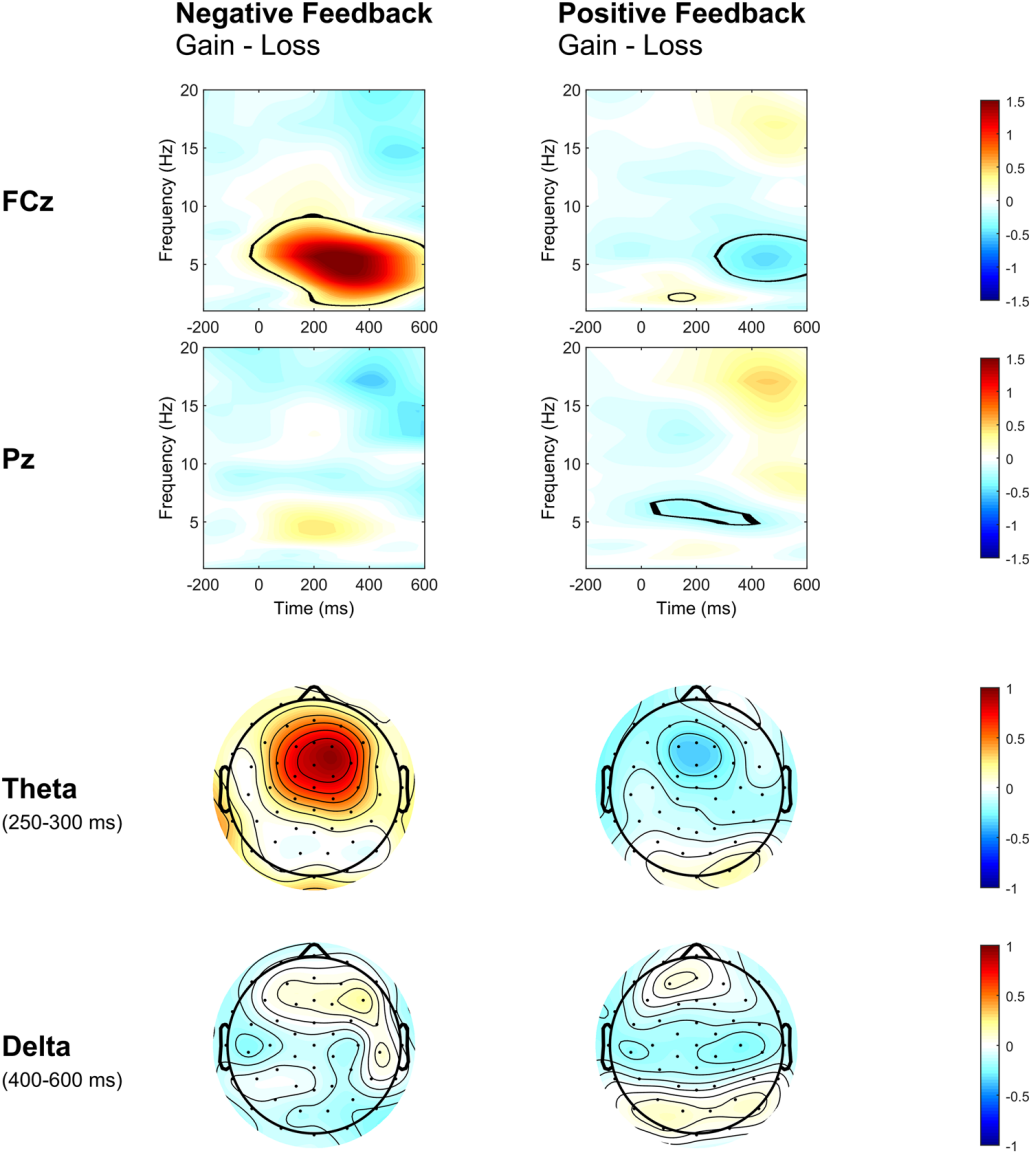
Difference in feedback-locked time–frequency power (in dB) between contexts (potential gain minus loss avoidance) within correct trials with negative feedback and correct trials with positive feedback. Time courses of feedback-locked time–frequency power within correct trials with negative feedback (left column) and correct trials with positive feedback (right column) for the difference between potential gain and loss avoidance context are depicted for electrodes FCz and Pz. Black lines indicate areas of significant difference at *p* < 4.6630e^−4^ (FCz) and *p* < 1.1885e^−7^ (Pz) for negative feedback and at *p* < 1.9453e^−4^ (FCz) and *p* < 6.6510e^−5^ (Pz) for positive feedback. Topographical maps for theta band power difference (4–8 Hz) between 250 to 300 ms and delta band power difference (1–4 Hz) between 400 to 600 ms are depicted in the bottom panels.

The effects of motivational context were examined for frontocentral theta power following negative and positive feedback, respectively (see Fig. 7 for visualization of effects). For negative feedback, theta power (0–600 ms) was larger in the PG context. There were no effects at parietal electrodes or in the delta range. For positive feedback, frontocentral (240–600 ms) and parietal (28–399 ms) theta power were decreased in the PG context. At FCz, there was a significant increase of delta power (102–195 ms) in the PG context. Direct comparison of negative and positive feedback yielded larger theta as well as delta power for trials with negative feedback (see Supplementary Fig. 3 for effects of different feedback types).

## Discussion

The present study examined how neural correlates of PM are influenced by trial-based changes in motivational context. Changes in motivational context shifted the speed-accuracy trade-off towards higher accuracy in the LA and faster responses in the PG context. In the PG context participants could gain points, reacted faster and paid more attention to the target stimulus and the feedback (P2). During response processing, there was no difference between contexts. The negative feedback in the PG context induced the strongest responses (FRN, theta power) and we observed greater adaptation effects within the PG context. There was no context effect of negative feedback for delta power. We therefore identified multiple facets of adaptation in response to changing motivational context.

Behaviorally, slower RTs and overall higher accuracy in the LA context, as well as no change in accuracy between post-correct and post-error trials suggest an especially cautious response style with a higher focus on accuracy than on speed, fitting with predictions by Yee et al. ^15^. Unexpectedly, there was a decrease in non-decision time after errors: a decrease in this parameter is usually associated with faster stimulus encoding and RT^71,72^. Potentially, this leads to more accurate responding in the LA context, by increasing efficiency in stimulus encoding. An alternative explanation would be that after positive feedback there is an increase in non-decision time to actively avoid the aversive outcome, which could be connected to higher P3a and P3b in the LA context. For the PG context, faster RT and higher error rate, as well as an increase in accuracy on post-error trials in the PG context, suggest more approach-oriented responding, also in line with Yee et al.^15^. There also was a difference in decision boundary between post-error and post-correct trials with positive feedback and a PIA effect only found for the PG context. Accuracy and decision boundary were both lower on post-correct trials. Higher decision boundaries are associated with more evidence accumulation before the response and therefore higher accuracy^73^, indicating less cautious responding in the PG context. However, we did not find the expected difference for drift rate. These effects fit into the EVC theory and its extensions^14,15,74^, the effects of loss aversion as proposed by Sokol-Hessner and Rutledge^13^, and the hierarchical reinforcement learning theory of anterior cingulate cortex function^34^. These models propose more investment of cognitive control when the expected reward is high, and more response caution for potential losses, weighing the cost of effort. Expected reward and efficacy are, according to these theories, weighed against each other and influence behavioral and neural signatures of control allocation and incentive processing^75^.

We observed larger P2 amplitudes in the PG context during stimulus processing. The P2 is associated with selective attentional processes engaged in evaluating the behavior relevant for adaptive performance based on the stimulus, and important for optimizing the current performance based on that evaluation^76–78^. Similarly, the P3a was also larger in the PG context, which is also associated with attentional processes^79,80^. This fits with the self-reported attentional bias toward the PG context cue. Together with behavioral findings these observations suggest that participants attributed higher motivational salience towards the PG context.

We found no difference between context for ERPs during response processing (i.e., ERN, CRN and Pe), which is contrary to results from some previous studies that reported changes in ERN amplitude between different motivational contexts [e.g.^20,25^]. However, this aligns with other research reporting no difference between motivational contexts^26^. Each of these studies used different tasks and incentive manipulations: be it presenting the contexts in block designs^25,26^ vs. trial-wise with the stimulus itself indicating the context^20^; or having deterministic feedback^20,26^ vs. introducing some uncertainty to the performance feedback by including RT in performance evaluations^25^. However, there is no apparent pattern in study designs that could explain these differences in results. Changes in response processing depending on motivational context may also express themselves more in the form of general enhancements compared to non-incentive contexts [see^23,24,26,27^]. Potentially, effects may have also been obscured by participants relying more on external feedback than their motor cues because of the implemented deadline. However, it has been shown in a similar task without motivational contexts that incorrect button presses, but not timing errors (analogue to our task defined by an individual deadline), affected the ERN^81^; similarly, incorrect button presses influenced the ERN, whereas correct decisions with an action execution worse than expected (spatial accuracy errors, similar to correct trials with slow responses resulting in timing errors) did not^82^. Importantly, in our analysis, response errors were defined as incorrect button presses, whereas slow responses resulting in timing errors and negative feedback were not included in ERN analysis, making a reliance on motor cues rather than external feedback more likely. Additionally, the processing of deviations between intended and actual response may only be changed for different motivational contexts depending on individual dispositions like compulsivity, conscientiousness and impulsivity^23,26,77,83^. Further studies are needed to disentangle these connections.

Our findings suggest that feedback processing is influenced by context. Negative feedback elicited higher amplitudes for the FRN and P3a in the PG context, whereas positive feedback elicited more positive P3a and P3b amplitudes in the LA context. Perhaps the most influential hypothesis on the FRN is the account that the FRN might reflect a hierarchical reinforcement learning process^34,51^: the appropriate task to implement is determined by the anterior cingulate cortex (ACC), then task execution is motivated by applying control over action-production systems, like the basal ganglia, which compute prediction errors (PEs) representing the deviation between expectation and outcome. The PEs are then transmitted to the ACC. The level of control is regulated according to rewards received^35,36^. Interestingly, the ACC is also proposed to learn reward values of task contexts^33,35^. RPE signals have been suggested to be modulated by feedback expectancy, have opposite signs for unexpected positive relative to unexpected negative events, and there is increasing evidence for the FRN being an RPE signal^32,33^. This partially corresponds to our results, with FRN amplitudes being larger for negative feedback. However, the context effect in our study cannot be fully explained by RPE: negative feedback in the PG and the LA context had comparable PE (relative loss of 40 points), and the FRN after response errors was higher in the PG context, despite response errors occurring more frequently. However, there are alternative explanations suggesting the FRN may not reflect the RPE but rather expectancy or surprise^37,38^. Surprise signals are larger to unexpected relative to expected events, irrespective of outcome valence^84^. The context effect on FRN in our study can therefore not be fully explained by either: the FRN was larger in the PG context although the frequency of negative feedbacks was similar for both conditions. Potentially, context may influence subjective expectancy of rewarding feedback and thereby affect FRN amplitude: participants may have perceived the PG context as more controllable, and may have consequently had a higher expectancy of positive feedback^85,86^. All this supports accounts proposing that the FRN is likely the result of multiplexed influences, presumably RPE, the sign or valence of the outcome (irrespective of magnitude), expectancy or surprise, incentive domain (i.e. alternative outcomes) and motivational salience^39,40^. Stewardson and Sambrook^40^ propose that the feedback valence, which they define as the sign of the RPE sensitive to the current trial and therefore context dependent, likely overlaps with the motivational salience which results in apparently stronger encoding of valence within the gain domain. The FRN has been shown to be sensitive to the dimension of feedback valence that is most salient [e.g.^32,87^]. This could explain higher FRN amplitudes for negative feedback within the PG context, even though quantitative RPE and expectancy were matched between PG and LA context. Supporting this, there is a reported attentional bias towards stimuli currently or previously associated with reward^88–90^.

Additionally, frontal midline theta power after negative feedback was higher for the PG vs. LA context, there was no context effect for delta power. Frontal midline theta power has been associated with the exertion of cognitive control during PM^58,59^. Consistent with this idea, incentive context was shown to influence perceived control: when outcomes were framed as a potential loss, participants significantly lowered their subjective value of control^85^. The perception of control, in turn, may increase the subjectively estimated probability of a reward, and also how much a reward is subjectively worth. Higher perceived control has been shown to elicit larger FRN and larger differences between reward and non-reward FRN^86,91^. Consequently, in the PG context the subjective value of positive feedback may have been increased, as may have the subjectively estimated probability of a rewarding feedback: Even though response errors were more frequent in the PG context, the FRN after response errors was still higher compared to the LA context. This process would shift the expectancy of gains, which in turn could explain the FRN amplitude being more sensitive to negative feedback in the PG context.

Limitations of the current study include that the task did not have a non-incentive context. There is evidence that ERPs during response processing are sensitive to incentive contexts as compared to non-incentive ones^23,27^. Aside from that, context effects might be obscured in tasks with a trial-by-trial compared to a block-wise manipulation because the tasks require constant attention^92^. Thus, future studies should consider multiple feedback manipulations. Additionally, we had a small number of trials for robust parameter estimation of some of the DDMs^93^ and introducing response deadlines potentially decreased RT variability, also hindering the detection of differences. We also did not prompt participants to report the prioritization of speed and accuracy separately for motivational context, which would have potentially corroborated the behavioral findings.

To summarize, stimulus- and feedback-locked brain activity was significantly influenced by trial-based manipulation of PG and LA motivational context. Our results suggest that more attention is allocated to the PG context, as evidenced by the stimulus- and feedback-P2, and the stimulus-P3a. Motivational context also affected behavioral performance and feedback processing: In the PG context, responding fast is prioritized, whereas in the LA context, responding accurately is. Negative feedback elicited more negative FRN in the PG context, while positive feedback elicited more positive P3a and P3b in the LA context, possibly signaling for maintenance of response speed and caution. The FRN effect in the current study is likely influenced by motivational salience and subjective probability of rewarding feedback. PG and LA context did not influence response-locked brain activity, although we could not compare the ERPs to a non-incentive condition. Our results demonstrate that motivational context has a significant influence on behavioral responding and performance monitoring during both the stimulus and feedback processing stages.

## Methods

### Sample

One hundred and forty participants were recruited from the general population in the Dresden, Germany, area. Seven participants committed more than 40% errors across all trials, two had a significant number of random button presses, and another participant discontinued the assessment. They were therefore excluded from further analyses. The final sample consisted of 130 participants (58.5% female; M = 25.88 years, SD = 5.66), 120 participants (92.3%) had completed advanced education degrees, 9.2% reported past mental health problems. Participants self-identified as of mainly European (95.4%) or of mainly Asian (4.6%) ancestry. All participants were native speakers of German, reported no history of head trauma or neurological disease and had normal or corrected-to normal vision. Participants were not included if they reported a history of bipolar disorder; emotionally unstable personality disorder; psychotic episodes; severe alcohol use disorder; currently met the criteria for an eating disorder or severe episode of major depression; reported taking psychotropic substances within the past 3 months; reported a lifetime use of illicit substances of more than twice a year; and lifetime use of cannabis of more than twice a month.

The study was conducted in accordance with the ethical guidelines of the Declaration of Helsinki. The ethics committee at the University Hospital Carl Gustav Carus, Technische Universität Dresden approved study procedures (EK 372092017). All participants gave informed consent. This sample has been analyzed before with a different focus of analysis [see^62^].

### Procedure, measures and tasks

#### Procedure

Participants took part in two sessions in the lab, and between those sessions a week of ecological momentary assessment. The effect of reward and punishment on PM-related brain activity was assessed during a monetary incentive flanker task using EEG during the second session. The EEG session took place a minimum of 8 days after the first session. During both sessions, participants performed other tasks, which are not part of this report.

#### Monetary incentive flanker task

A modified version of the arrow-version of the Eriksen flanker task, with a potential gain (PG) and a loss avoidance (LA) incentive context, was employed^60,61^. Participants had to respond as quickly and correctly as possible using a left or right button, according to the direction of the centrally presented target arrow. The target stimulus appeared for 30 ms, with a delay of 100 ms relative to the onset of the surrounding flanker arrows. In 50% of the trials, the target stimulus pointed in the opposite direction (incongruent) as the flanker arrows. In the other 50% of the trials, the target pointed in the same direction (congruent). Each trial started with an incentive cue, signaling potential gain or loss of 40 points in the current trial. The incentive cue, a red (LA) or green (PG) frame surrounding a fixation cross, was presented for 500 ms. The frame remained visible for the duration of the trial. After response, feedback on task performance was presented: negative feedback was presented for incorrect and the slowest 20% of the correct responses and positive feedback for the remaining correct responses. In the LA context (50% of the trials) negative feedback was associated with a loss of 40 points and positive feedback with loss omission (0 points). In the PG context positive feedback resulted in a gain of 40 points and negative feedback in reward omission (0 points). An adaptive deadline based on individual performance and response time determined the response deadline, in order to approach a rate of 20% negative feedback for each context (PG context: M = 18.6%, SD = 2.3%; LA context: M = 19.6%, SD = 2.3%). After a response interval of 900 ms following target onset or 600 ms after response, performance feedback was presented for 800 ms. Participants could earn a bonus of up to 5 EUR, depending on task performance and points earned. The task included 640 trials of 2.53–2.75 s duration and an additional training block of 40 trials. Task duration was approximately 25 min. After task completion, participants were asked, on a visual analogue scale, how important it was to them to respond accurately, how important it was for them to respond fast, and how much attention they paid to the red and the green frame, respectively. The task was presented using Presentation 19.0 (Neurobehavioral Systems Inc., Berkeley, CA, USA). See Fig. 1a for a schematic depiction of the task.

### Psychophysiological recording and data reduction

The EEG was continuously recorded using elastic EEG caps with 63 Ag/AgCl electrodes at equidistant locations (EasyCap GmbH, Herrsching-Breitbrunn, Germany) and two 32-channel BrainAmp amplifiers (Brain Products GmbH, Munich, Germany) at a sampling rate of 500 Hz. Impedances were kept below 10 kOhm. Eye movement was captured using two external electrodes placed below the left and right eye together with electrodes above both and lateral to both eyes mounted into the cap. Ground and reference electrodes were placed next to Fz (at AFF1h and AFF2h, theta/phi spherical coordinates: − 58/78 and 58/78). The EEG was high and low pass filtered with cutoffs of 0.1 and 30 Hz, respectively, and epoched from –500 to 2000 ms relative to target stimulus onset. Epochs with artifacts were rejected automatically based on signal deviations greater than 5 SD of the mean probability distribution on any single channel or the whole montage. Remaining epochs were demeaned and submitted to adaptive mixture independent component analysis (AMICA), implemented in EEGLAB 14.1.2^94^. Independent components reflecting ocular or cardiovascular artifacts were removed manually and EEG data were re-referenced to common average reference. Subsequently, target stimulus-locked, response-locked and feedback-locked epochs from − 500 to 1000 ms were created. The average EEG activity 200–0 ms prior to stimulus, response and feedback was used as baseline, respectively. Offline analyses were performed using EEGLAB 14.1.2^94^ and MATLAB 2018b^95^. Data were then further analyzed using multiple robust single-trial regression analyses [e.g.^68,96^].

### Data analysis

#### Behavioral data

We employed two multiple robust regression models onto each participant’s single-trial accuracy and log-transformed reaction time (RT) to determine predictors of influence^65^. The RT model was specified as follows: *log*(*RT)* = *b*_*0*_ + *congruency x b*_*1*_ + *previous accuracy x b*_*2*_ + *context x b*_*3*_ + *accuracy x b*_*4*_ + *trial x b*_*5*_ + *e*. The logistic accuracy model was specified by: *accuracy* = *b*_*0*_ + *congruency x b*_*1*_ + *previous accuracy x b*_*2*_ + *context x b*_*3*_ + *trial x b*_*4*_ + *e*. The predictors are: *congruency* (congruency between flanker and target; − 1 = congruent, 1 = incongruent), *previous accuracy* (accuracy of the immediately preceding trial; − 1 = correct, 1 = error), *context* (incentive context of the current trial; − 1 = LA, 1 = PG), *accuracy* (of the current trial; − 1 = correct, 1 = error) and *trial* (trial number, reflecting the time on the task). *Trial* mainly served to control for unspecific effects of task duration (e.g. adjustments of speed-accuracy trade-offs over the task or blocks, or fatigue). Individual *t*-values per regressor were tested via two-sided *t*-tests against zero, on group level, all *p*-values were Bonferroni corrected. Follow-up analyses are shown in respective figures (Fig. 1).

Because the self-report data were not normally distributed as determined by the Shapiro–Wilk test of normality (of the differences), we analyzed differences between behavioral measures using the Wilcoxon signed rank test^97,98^. *P* values were adjusted for multiple comparisons using the procedure proposed by Benjamini and Yekutieli^99^.

#### Drift–diffusion modelling

Features of the multi-stage sequential sampling models and general fitting procedures were exactly as described by Fischer et al.^65^ and Kirschner et al.^67^. The description is adapted from therein. We used a multi-stage sequential sampling model to simulate participants' RT and accuracy distributions and the decision process giving rise to these. The model approximates the decision process as a single decisions variable which reflects both decision options. We previously demonstrated that this approximation is valid and compatible with an independently measured neural decision signal (i.e., lateralized movement selective beta power (BPL))^65,67^. The discrete diffusion model simulates decisions as a Wiener process with stepwise increments according to a Gaussian distribution with mean ***v*** (called drift rate) and variance ***s*** on every trial. Here, ***v*** reflects the speed of evidence accumulation and ***s*** the system’s noise, which scales all other parameters and is usually fixed (here to 0.1). The step size for all models was set to 1 ms. Responses are triggered when the diffusion reaches a criterion (boundary, determined by the free parameter ± ***a***). We assumed that there was no bias in response selection over the task, because we had an equal number of left and right responses. However, individual trials were allowed to start with a random bias towards one response (start-point variability, parameter ***sz***). We used symmetrical boundaries which were defined as left-hand responses when the positive boundary was reached first, and as right-hand responses when the negative boundary was reached first (see Fig. 2a for more details). The non-decision time was modelled as another free parameter ***T***_***er***_. The stages of the model per trial were defined as a zero-mean baseline until flanker onset, a noisy diffusion with v = 0 during the non-decision time, a diffusion driven by the flanker direction between flanker and target onset (100 ms) with drift = vt × ft, and the target phase thereafter with drift = ***vt*** and the direction of the target. Single-trial values ***vt*** and ***ft*** were determined according to Gaussian variance parameters ***sv*** and ***sf***. For display purposes, in Fig. 2a, we modelled a consecutive return of the decision variable to baseline like an Ornstein–Uhlenbeck process. To speed up the model fitting procedure, we did neither simulate baseline periods nor return to baseline during the fitting because these have no effect on model predictions. We compared four different variants of the DDM by fitting their parameters to RT and accuracy data observed in the group of 130 participants using quantile maximum likelihood statistics^100^ and differential evolution algorithms^101^. Additionally, we used a mixture model assuming 2% outliers that were distributed uniformly over the full range of RTs in correct and error responses. This downweighs the impact of possible outliers on model parameters. DDM model 1 used the same drift rate during flanker and target processing, thus not allowing for suppression of distractors (parameter ***f*** fixed to 1). DDM model 2 fixed parameter ***f*** which suppressed flanker processing when below the value of 1. DDM model 3 furthermore included trial-by-trial variance in ***f*** modelled as a zero-mean Gaussian distribution with variance ***sf***. The fourth DDM model extended DDM model 3 to allow for a dynamic decision boundary collapse according to a Weibull distribution scaled by the free parameter ***k***. Here, the dynamic boundary ***u*** at time ***t*** was calculated as follows:

$${u}_{t}=a-\left(1-{\mathrm{exp}-\left(\frac{t}{k}\right)}^{s}\right)*\frac{a}{2}$$

In this equation, ${\varvec{a}}$ represents the initial boundary value and ${\varvec{k}}$ scales the Weibull distribution. The shape parameter ${\varvec{s}}$ was fixed at 3, to impose a “late collapse” decision strategy.

For model fitting in all iterations, we applied the following hard priors which can be seen as boundary parameters: **v** [0.01–8.5], **sv** [0–1.5], **a** [0.01–0.45], **sz** [0.05–0.3], ***T***_***er***_ [0.1–0.4], **st** [0–2], **f** [0.1–1.5], **sf** [0–1.5], **k** [10e-4–3].

For model comparison, we first computed approximated BIC values (aBIC; akin to White et al.^102^). Next, we used these individual aBIC values to compute protected exceedance probabilities, which gives the probability that one model was more likely than any other model of the model space Rigoux et al.^103^. As DDM4 provided the best fit to the data, we used this model to investigate model parameters when this model was fit separately to post-error and post-correct trials with positive and negative feedback, respectively, across all contexts and additionally within the PG and LA context. For this analysis, we fixed the variance parameters (sv, sz, st, sf) to the group mean to facilitate convergence.

To simulate the temporal evolution of the modelled diffusion signal depicted in Fig. 2a we used the mean maximum likelihood parameters from the group fit obtained for DDM4. For this simulation, we computed 5000 simulated trials.

#### EEG analyses

Multiple robust regression^104^ was employed to build a general linear model (GLM) and regress behavioral and task parameters on single-trial EEG activity at each electrode and time point. The resulting individual *b* values were standardized by their SDs before averaging across subjects, to penalize the regression model in case of multicollinearity of predictors and ensure comparability between predictors.

##### Stimulus-locked analyses

The model for target stimulus-locked EEG included only correct trials and included a regressor coding the incentive context of the current trial (loss/gain), the interaction between context and congruency of the current trial, the congruency (congruent/incongruent) of the current trial, the accuracy of the previous trial (correct/error) and log scaled RT.

*Response-locked analyses.* We built a model for response-locked EEG, which only included incongruent trials. The model included a regressor coding the accuracy of the current trial (correct/error), incentive context of the current trial (loss/gain) and the interaction between accuracy and context of the current trial. Log scaled RT of the current trial and response hand (left/right) were included as additional regressors.

##### Feedback-locked analyses

We focused our data analysis on feedback after correct responses, as only feedback after correct responses carried new information and erroneous responses deterministically elicited negative feedback. The first feedback model included the regressors coding the incentive context of the current trial (loss/gain), the feedback valence (negative/positive), and the interaction between feedback and context. To follow-up the interaction, we built two additional models for correct trials with corresponding positive feedback and for correct trials with negative feedback, respectively. Both models included the incentive context of the current trial (loss/gain) as the only regressor. See the Supplement for an analysis of error trials and corresponding negative feedback, a cue-locked analysis, as well as the effect of feedback type within the respective context.

The standardized *b* values resulting from the models were tested using two-tailed one-sample t-tests, for FCz and Pz. The tests were done for each data point across subjects. The resulting *p* values were adjusted for multiple comparisons using the false discovery rate (FDR) procedure proposed by Benjamini and Yekutieli^99^. We set a criterion value of 0.001 to minimize the chance of false positive results. The FDR procedure by Benjamini and Yekutieli^99^ has been found to facilitate good control of the family-wise error rate (FWER) in EEG data^105^. As FDR does not provide sound local control of the FWER, FDR was applied per model and electrode. H_0_ was rejected for *p* < 2.2654e^−4^ (FCz) and *p* < 3.2189e^−4^ (Pz) in the stimulus-locked, for *p* < 2.8827e^−4^ (FCz) and *p* < 3.4804e^−4^ (Pz) in the response-locked model, for *p* < 3.7708e^−4^ (FCz) and *p* < 2.7407e^−4^ (Pz) in the first feedback-locked model, for *p* < 1.9288e^−4^ (FCz) and *p* < 4.2192e^−4^ (Pz) in the feedback-locked model for correct trials with corresponding positive feedback and for *p* < 2.2664e^−4^ (FCz) and *p* < 2.1000e^−5^ (Pz) for the feedback-locked model for correct trials with negative feedback.

##### Time–frequency analysis of feedback

We also analyzed feedback-locked activity in the time–frequency domain, to investigate whether possible differences between contexts were due to theta- or delta-band activity. Analyses were conducted according to recommendations by Cohen^106^. Data were epoched around the feedback onset (− 1000 to 2000 ms). The interval between − 500 to − 200 ms was used for baseline correction. Time–frequency decomposition was done separately for each combination of feedback type and context. EEG time series in each epoch were convolved with a set of complex Morlet wavelets, defined as a Gaussian-windowed complex sine wave: ${\mathrm{e}}^{-\mathrm{i}2\mathrm{\pi ft}}{e}^{ \frac{-4{\mathrm{log}(2)t}^{2}}{{h}^{2}}}$, where t is time, f is frequency, which increased from 1 to 20 Hz in 20 logarithmically spaced steps, and h defines the full-width at half-maximum, which ranged from 600 to 300 ms with 20 logarithmically spaced steps^107^. Power was normalized by conversion to a decibel (dB) scale (10 ∗ log10[power(t)/power(baseline)]), to allow direct comparison of effects across frequency bands. Each epoch was then cut to − 200 to 600 ms. Epochs were then averaged for each context, feedback type and participant. The differences between the PG and LA contexts were tested using two-tailed one-sample t-tests, for FCz and Pz. The tests were done for each data point across subjects, within a time window from 0 to 600 ms and frequencies between 1 and 8, to include delta and theta bands. Differences between contexts were tested for negative and positive feedback separately. The resulting *p* values were adjusted for multiple comparisons using the FDR procedure proposed by Benjamini and Yekutieli^99^. We set a criterion value of 0.001 to minimize the chance of false positive results. H_0_ was rejected for *p* < 4.6630e^−4^ (FCz) and *p* < 1.1885e^−7^ (Pz) for the comparison of contexts in negative feedback, and for *p* < 1.9453e^−4^ (FCz) and *p* < 6.6510e^−5^ (Pz) for the comparison of contexts in positive feedback.

Statistical analysis of EEG and behavioral data was performed in MATLAB 2018b^95^ and R 4.2.0^108^.

### Supplementary Information

Supplementary Information.

## Data Availability

The data that support the findings of this study are available on OSF (https://osf.io/h6ju7/) and from the corresponding author on request.
