# Supplementary material for: Unraveling the influence of trial-based motivational changes on performance monitoring stages in a flanker task
Source: Sci Rep. 2023 Nov 6;13:19180. doi: 10.1038/s41598-023-45526-0 (PMC10628251; doi:10.1038/s41598-023-45526-0)
Supplement: Supplementary file 1 — Supplementary Information. [file 41598_2023_45526_MOESM1_ESM.docx]

**Supplementary Note**

**Unraveling the Influence of Trial-Based Motivational Changes on Performance Monitoring Stages in a Flanker Task**

Rebecca Overmeyer^1^*, Hans Kirschner^2^, Adrian G. Fischer^3^, Tanja Endrass^1,4^

1 Faculty of Psychology, Technische Universität Dresden, Dresden, Germany; 2 Institute of Psychology, Otto-von-Guericke University; 3 Department of Education and Psychology, Freie Universität Berlin, Berlin, Germany; 4 Neuroimaging Center, Technische Universität Dresden, Dresden, Germany

*Corresponding Author

Rebecca Overmeyer

Technische Universität Dresden

Institute of Clinical Psychology and Psychotherapy

Chair for Addiction Research

Chemnitzer Straße 46a

01187 Dresden

[rebecca.overmeyer@tu-dresden.de](mailto:rebecca.overmeyer@tu-dresden.de)

Tel.: +49 351 463 39860

**Behavioral effects**

Behavioral effects in the current study match those typically observed in a flanker task. Logistic single-trial regression on accuracy (GLM1) and single-trial regression on reaction time (RT; GLM2) were used to analyze behavioral data (see Fig. 1 in main text).

On incongruent trials, participants were significantly less accurate (main effect *congruency*, *t*_129_ = 17.08, *p* < 10^-33^, two-sided t-test against zero, all p-values are Bonferroni corrected). Accuracy was also lower in the gain context (main effect *context*, *t*_129_ = 10.19, *p* < 10^-16^). After error commission, accuracy increased on the following trial (post-error increase in accuracy, main effect of *last accuracy*, *t*_129_ = 4.63, *p* < 10^-4^). This effect was modulated by congruency of the current trial (*congruency x last accuracy*, *t*_129_ = -4.94, *p* < 10^-4^), as well as context of the current trial (*context x last accuracy*, *t*_129_ = -2.68, *p* = 0.049).

RT was lower on congruent trials (main effect *congruency*, *t*_129_ = 47.93, *p* < 10^-82^), confirming the presence of an interference effect [1]. RT was higher for the loss context (main effect *context*, *t*_129_ = -4.92, *p* < 10^-4^). Error RTs were faster than correct RTs (main effect of *accuracy*, *t*_129_ = -3.65, *p* = 0.004). This was modulated by congruency (*congruency x accuracy*, *t*_129_ = -25.55, *p* < 10^-43^) and context (*congruency x accuracy x context*, *t*_129_ = -3.31, *p* = 0.011). Following error commission, RTs were slower (main effect *last accuracy*, *t*_129_ = 4.27, *p* < 10^-3^), confirming post-error slowing (PES). Post-error slowing (PES) was modulated by motivational context and congruency (*congruency x last accuracy*, *t*_129_ = -3.01, *p* = 0.028, *congruency x last accuracy x context*, *t*_129_ = 3.55, *p* = 0.005).

See Table 1 for descriptive statistics of behavioral data.

| **Table 1**  Task performance and measures of performance monitoring (means and standard deviations) in the Monetary Incentive Flanker Task. | | | | | | |
| --- | --- | --- | --- | --- | --- | --- |
|  | **All trials** | | **Gain trials** | | **Loss trials** | |
|  | *M* | *SD* | *M* | *SD* | *M* | *SD* |
| *Correct trials (ms)* |  |  |  |  |  |  |
| Correct incongruent RT | 381 | 32 | 381 | 33 | 380 | 32 |
| Correct congruent RT | 291 | 32 | 289 | 32 | 293 | 32 |
|  |  |  |  |  |  |  |
| *Trials around errors (ms)* |  |  |  |  |  |  |
| Error RT | 260 | 29 | 258 | 29 | 262 | 30 |
| Post-correct RT | 325 | 35 | 323 | 35 | 326 | 35 |
| Post-error RT | 331 | 39 | 328 | 41 | 335 | 38 |
| Pre-error RT | 308 | 33 | 307 | 33 | 310 | 34 |
|  |  |  |  |  |  |  |
| *Trials after feedback (ms)* |  |  |  |  |  |  |
| Post-positive RT | 324 | 35 | 323 | 35 | 325 | 35 |
| Post-negative RT | 326 | 38 | 323 | 39 | 328 | 38 |
|  |  |  |  |  |  |  |
| *Accuracy (%)* |  |  |  |  |  |  |
| Error rate | 16.66 | 8.21 | 17.95 | 8.55 | 15.36 | 8.15 |
| PEA | 86.72 | 8.49 | 86.56 | 8.87 | 86.84 | 9.35 |
| PCA | 85.90 | 7.38 | 84.54 | 7.95 | 87.19 | 7.14 |
| *Note.* RT, reaction time; Error reaction times refer to all error trials; Pre-error reaction times refer to correct trials before error commission; Post-error reaction times refer to correct trials after error commission; Post-correct reaction times refer to correct trials after correct responses; Post-positive reaction times refer to correct trials after positive feedback in the correct trial before; Post-negative reaction times refer to correct trials after negative feedback in the correct trial before; PEA, Post Error Accuracy; PCA, Post Correct Accuracy. | | | | | | |

**Drift-diffusion modelling**

| *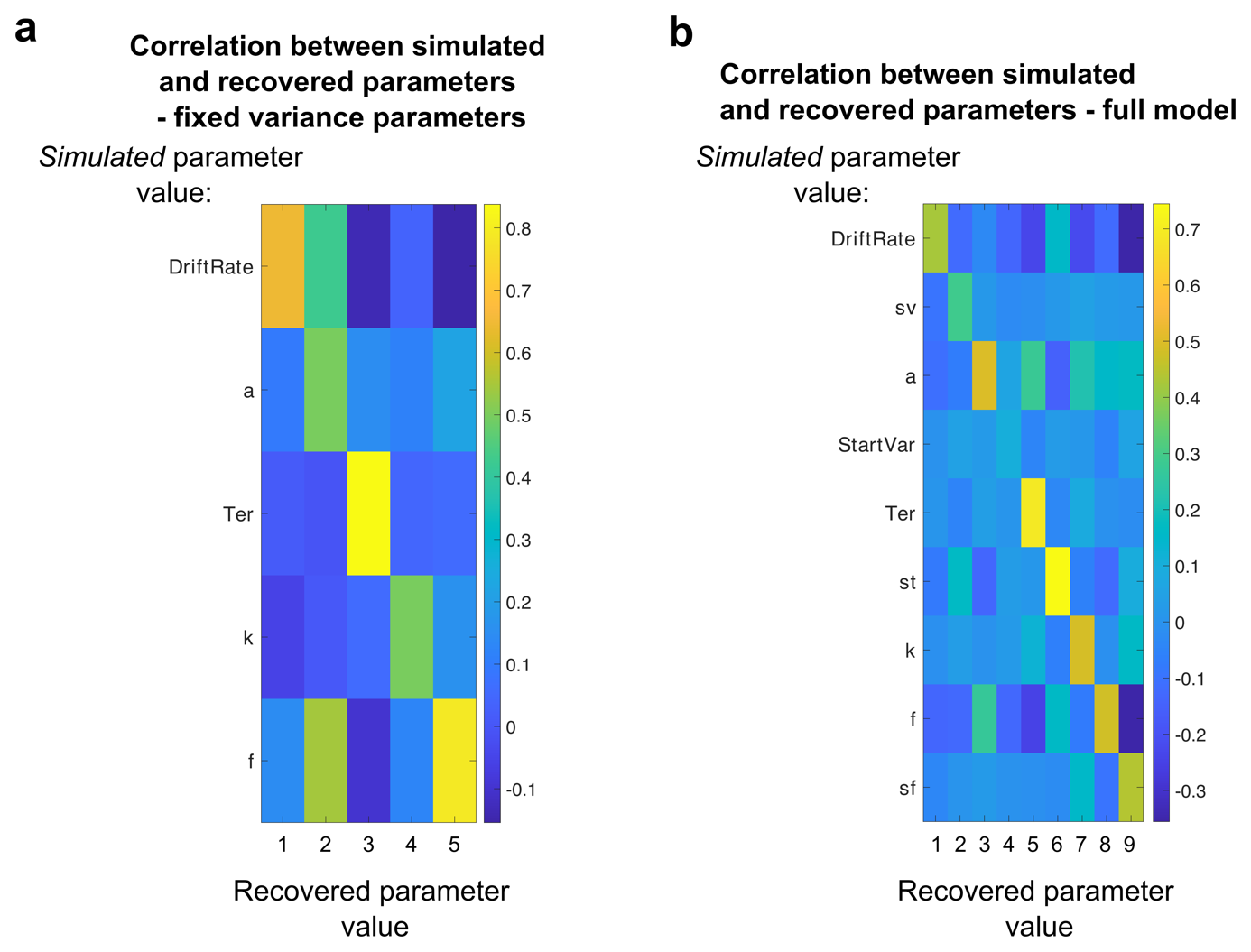* |
| --- |
| FIGURE 1 \| Parameter recovery. Here, we show parameter recovery analyses for which we randomly drew model parameters out of a Gaussian distribution with mean and variance equal to the observed fitted parameters across the whole group to reduce parameter value combinations that were extremely unlikely to occur in human data. We simulated 1.000 parameter combinations and used the same differential evolution algorithm to recover the fitted models. Models that produced no errors at all or for which constraints were not met, were discarded from the analysis. As in the human data, we used 5.000 trials per simulation. **(a)** Correlation between simulated and recovered parameters with fixed variance parameters. Here, we fixed the variance parameters (sv, sz, st, sf) to the group mean. **(b)** Correlation between simulated and recovered parameters for the full model. Both analyses indicate that parameter values that were used to simulate data from the full feature DDM (ordinate) tended to correlate with the parameter values best fit to those synthetic datasets (abscissa). *Note:* the full feature DDM consisted of nine parameters: drift rate (*v*), variance in drift rates (*sv*), boundary (*a*), variance in start points (*sz*), non-decision time (*Ter,* reflecting visual processing and motor execution times), variance in *Ter* (*st*), bounds collapse (*k*), flanker suppression (*f*), and variance in *f* (*sf*). |

| 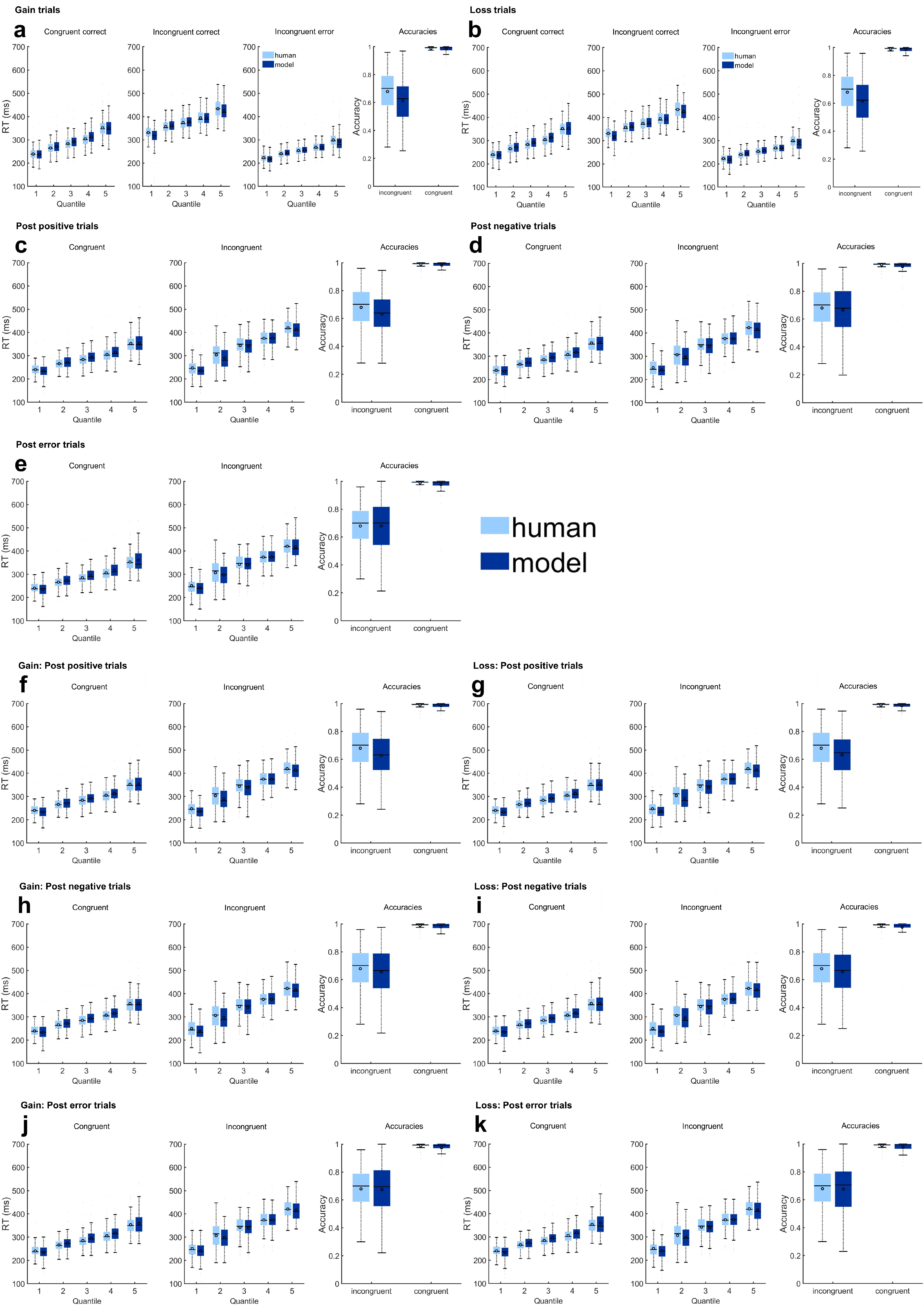 |
| --- |
| FIGURE 2 \| **a-k** Quantile fits of the winning model (dark blue) against human reaction time data (light blue), and model and human accuracy. Models were fit separately to all gain trials (**a**), loss trials (**b**), trials after correct responses with positive feedback (**c**), trials after correct responses with negative feedback (**d**), trials after errors (**e**), trials after correct responses with positive feedback with the current trial in the gain or loss context (**f**, **g**), trials after correct responses with negative feedback with the current trial in the gain or loss context (**h, i**), and trials after incorrect responses with the current trial in the gain or loss context (**j**, **k**). Overall, each model captured the behavior of the participants well. |

| **Table 2**  Maximum likelihood parameters of the winning DDM4 for all trial types. | | | | | | | | | |
| --- | --- | --- | --- | --- | --- | --- | --- | --- | --- |
|  | *v* | *sv* | *a* | *sz* | *T_er_* | *st* | *k* | *f* | *sf* |
|  |  |  |  |  |  |  |  |  |  |
| All trials | 5.74 | 0.66 | 0.37 | 0.18 | 0.19 | 0.73 | 2.23 | 0.59 | 0.48 |
|  |  |  |  |  |  |  |  |  |  |
| Gain trials | 2.52 | 0.66 | 0.21 | 0.18 | 0.20 | 0.73 | 1.62 | 0.76 | 0.48 |
| Loss trials | 2.54 | 0.66 | 0.20 | 0.18 | 0.20 | 0.73 | 1.61 | 0.76 | 0.48 |
|  |  |  |  |  |  |  |  |  |  |
| Post positive trials | 2.59 | 0.66 | 0.21 | 0.18 | 0.20 | 0.73 | 1.52 | 0.74 | 0.48 |
| Post negative trials | 2.65 | 0.66 | 0.22 | 0.18 | 0.20 | 0.73 | 1.37 | 0.72 | 0.48 |
| Post error trials | 2.73 | 0.66 | 0.23 | 0.18 | 0.19 | 0.73 | 1.53 | 0.69 | 0.48 |
|  |  |  |  |  |  |  |  |  |  |
| Gain trials |  |  |  |  |  |  |  |  |  |
| *Post positive trials* | 2.45 | 0.66 | 0.22 | 0.18 | 0.20 | 0.73 | 1.42 | 0.77 | 0.48 |
| *Post negative trials* | 2.43 | 0.66 | 0.22 | 0.18 | 0.20 | 0.73 | 1.41 | 0.76 | 0.48 |
| *Post error trials* | 2.60 | 0.66 | 0.23 | 0.18 | 0.19 | 0.73 | 1.53 | 0.71 | 0.48 |
|  |  |  |  |  |  |  |  |  |  |
| Loss trials |  |  |  |  |  |  |  |  |  |
| *Post positive trials* | 2.39 | 0.66 | 0.22 | 0.18 | 0.20 | 0.73 | 1.46 | 0.78 | 0.48 |
| *Post negative trials* | 2.55 | 0.66 | 0.22 | 0.18 | 0.20 | 0.73 | 1.41 | 0.73 | 0.48 |
| *Post error trials* | 2.82 | 0.66 | 0.23 | 0.18 | 0.19 | 0.73 | 1.58 | 0.66 | 0.48 |
| *Note.* The table shows the parameters obtained by fitting DDM4 to the individual subject data. For the fit to different trial types, variance parameters were fixed to the mean value across the group derived from the model for all trials. Individual fit parameters represent mean values. *v* = drift rate*, sv =* variance in drift rates*, a =* boundary*, sz =* variance in start points*, Ter =* non-decision time*, st =* variance in *Ter, k =* bounds collapse*, f =* flanker weighting*, sf =* variance in *f.* | | | | | | | | | |

**Supplementary EEG Analyses**

**Response-locked analysis of correct trials**

There was no evidence of an influence of context on the CRN, as there was no interaction between context and accuracy for the response-locked model.

**Supplementary analysis of feedback effects**

Differences between negative and positive feedback were examined for the correct trials in gain and loss avoidance contexts separately. The feedback models included the regressor coding the feedback valence (negative/positive). H_0_ was rejected for FCz (*p* < 3.0352e^-4^) and Pz (*p* < 3.8470e^-4^) in the gain context, and for FCz (*p* < 4.3459e^-4^) and Pz (*p* < 3.7909e^-4^) in loss avoidance context. Both models revealed a significant effect of feedback type on EEG activity at frontocentral electrodes corresponding to a time window associated with the P2 (156 to 216 ms for the gain context, and 158 to 230 ms for the loss avoidance context). The FRN was significantly larger for negative than positive feedbacks in the gain context (232 to 290 ms) as well as in the loss avoidance context (258 to 284 ms). The P3a was larger for negative feedback in both contexts (306 to 600 ms in the gain context, and 306 to 600 ms in the loss avoidance context). The same was true for the P3b (324 to 600 ms in the gain context, and 306 to 600 ms in the loss avoidance context). See Figure 3 for a visualization of results.

| 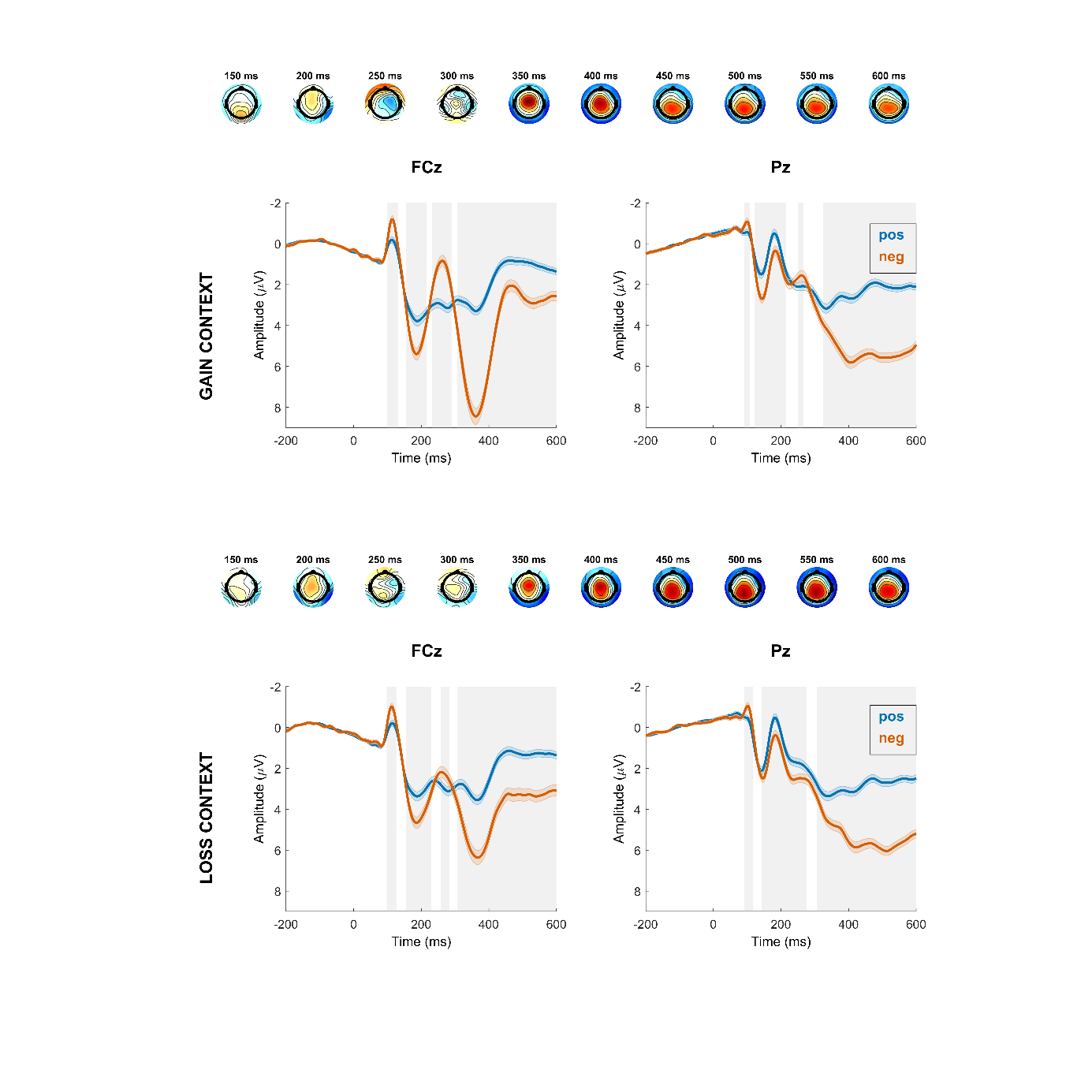 |
| --- |
| FIGURE 3 \| Time course of feedback-locked regression effects for significant regressor feedback (positive, negative) within correct trials in the gain context and correct trials in the loss context. The first and third row represent topographical maps of the associations between EEG activity and the feedback regressor within the respective trial type. Grand average event-related potential (ERP) waveforms at electrodes FCz and Pz are depicted in the second and fourth row, feedback-locked for correct trials within the gain or loss context. Shadows indicate the SEM. Gray shading behind the waveforms indicates significance at *p* < 3.0352e-4 (FCz) and *p* < 3.8470e-4 (Pz) for the gain context, and *p* < 4.3459e-4 (FCz) and *p* < 3.7909e-4 (Pz) for the loss context. |

**Time-frequency analysis of feedback effects within contexts**

Figure 4 represents time-frequency power within the different trial types.

Differences between feedback types were tested for the gain and loss avoidance contexts separately. The differences were tested using two-tailored one-sample t-tests, for FCz and Pz. The tests were done for each data point across subjects, within a time window from 0 to 600 ms and frequencies between 1 and 8, to include delta and theta bands. The resulting *p* values were adjusted for multiple comparisons using the FDR procedure proposed by Benjamini and Yekutieli [2]. We set a criterion value of 0.001 to minimize the chance of false positive results. H_0_ was rejected at FCz (*p* < 8.1052e^-4^) and Pz (*p* < 8.2877e^-4^) for the comparison of feedback type in the gain context, and at FCz (*p* < 8.7308e^-4^) and Pz (*p* < 8.0490e^-4^) for the comparison of feedback type in the loss avoidance context.

| **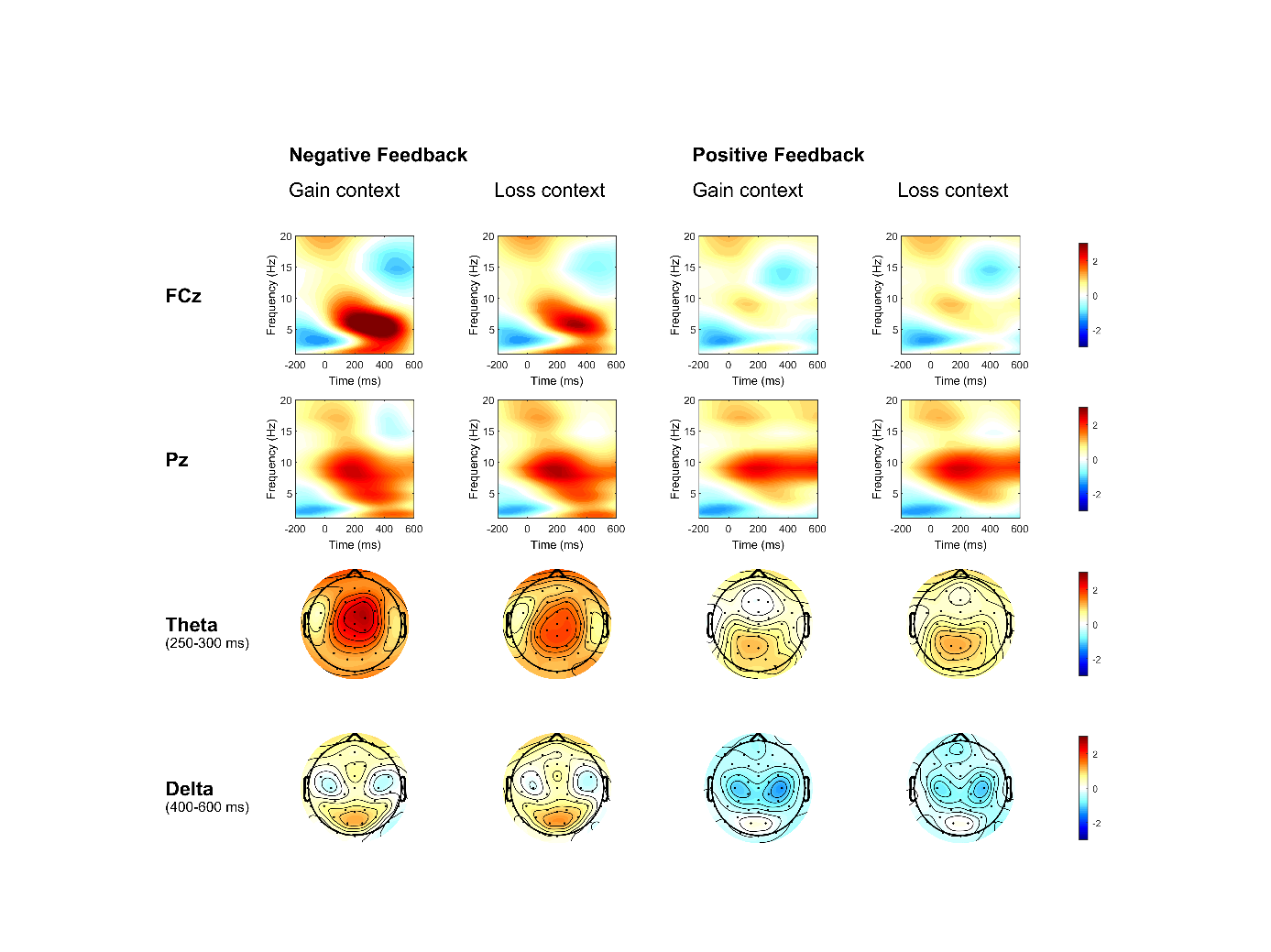** |
| --- |
| FIGURE 4 \| Feedback-locked time-frequency power (in dB) for gain and loss context within correct trials with negative feedback and correct trials with positive feedback. Time courses of feedback-locked time-frequency power are depicted at electrodes FCz and Pz. Topographical maps for theta band power (4-8 Hz) between 250 to 300 ms and delta band power (1-4 Hz) between 400 to 600 ms are depicted on the bottom panels. |

The comparison of negative and positive feedback exhibited a significant difference in theta power at both FCz (0 to 600 ms) and Pz (0 to 600), as well as a significant difference in delta power at FCz (0 to 600) and Pz (0 to 600). The comparison between feedback types within the loss avoidance context showed a significant difference in theta power for both FCz (12 to 600 ms) and Pz (202 to 503 ms) sites, as well as a significant difference in delta power at FCz (0 to 600) and Pz (0 to 600). In all comparisons the gain context yielded more power. See Figure 5 for a visualization of results.

| 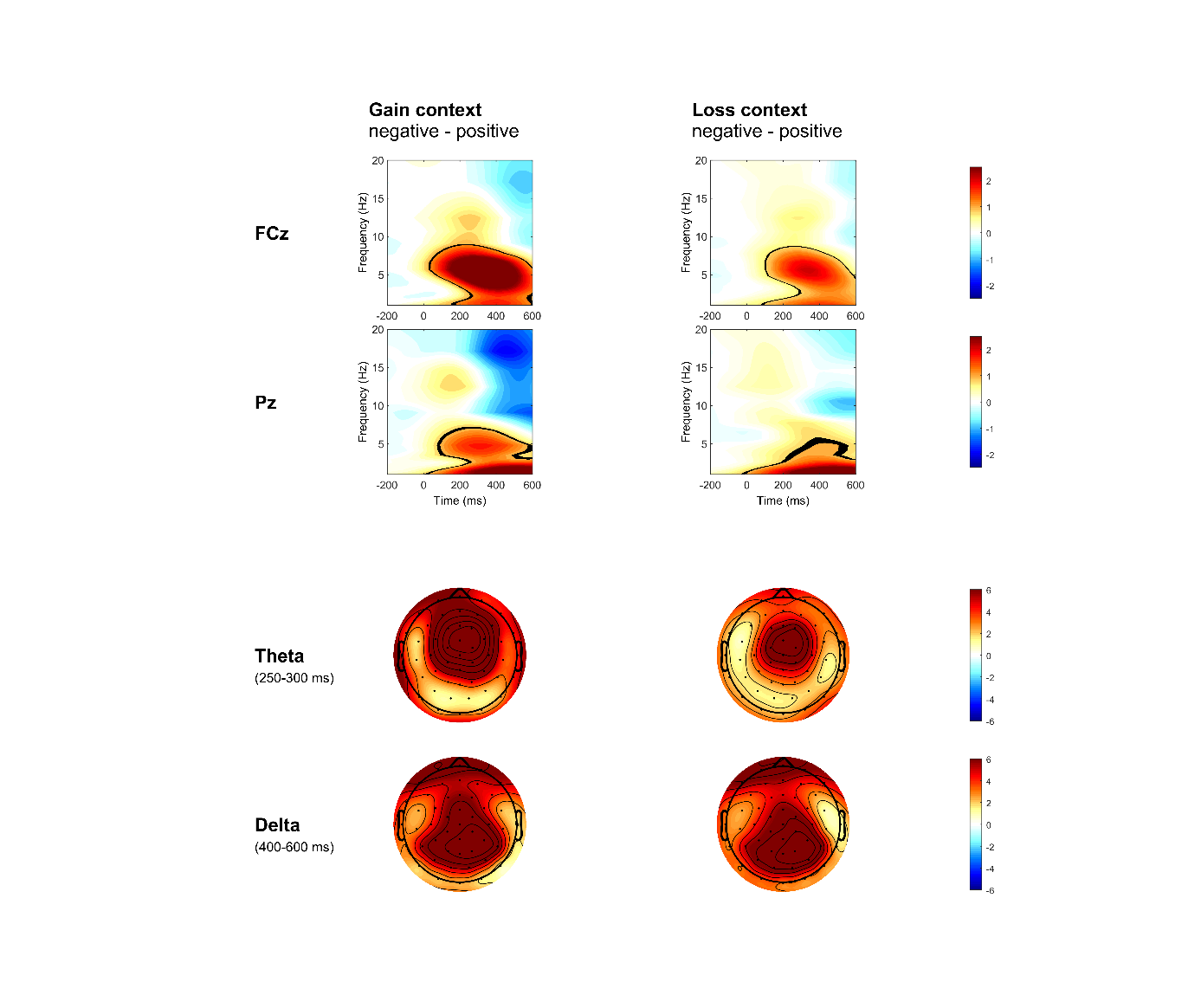 |
| --- |
| FIGURE 5 \| Difference in feedback-locked time-frequency power (in dB) between feedback types (negative minus positive) within the gain context and the loss avoidance context. Time courses of feedback-locked time-frequency power difference within correct trials in the gain context and correct trials in the loss context for negative and positive feedback are depicted for electrodes FCz and Pz. Black lines indicate areas of significant difference. Topographical maps for theta band power difference (4-8 Hz) between 250 to 300 ms and delta band power difference (1-4 Hz) between 400 to 600 ms are depicted on the bottom panels. |

**Feedback-locked analysis in error trials**

The model for feedback-locked EEG in error trials included a regressor coding the motivational context of the current trial (loss/gain). H_0_ was rejected at FCz (*p* = 8.0100e^-5^) and Pz (*p* = 2.4900e^-6^). The model revealed a significant covariation of context with feedback-locked EEG activity at the FCz in time windows corresponding to the FRN (216 to 250 ms and 276 to 310 ms). See Figure 6 for a visualization of results.

| 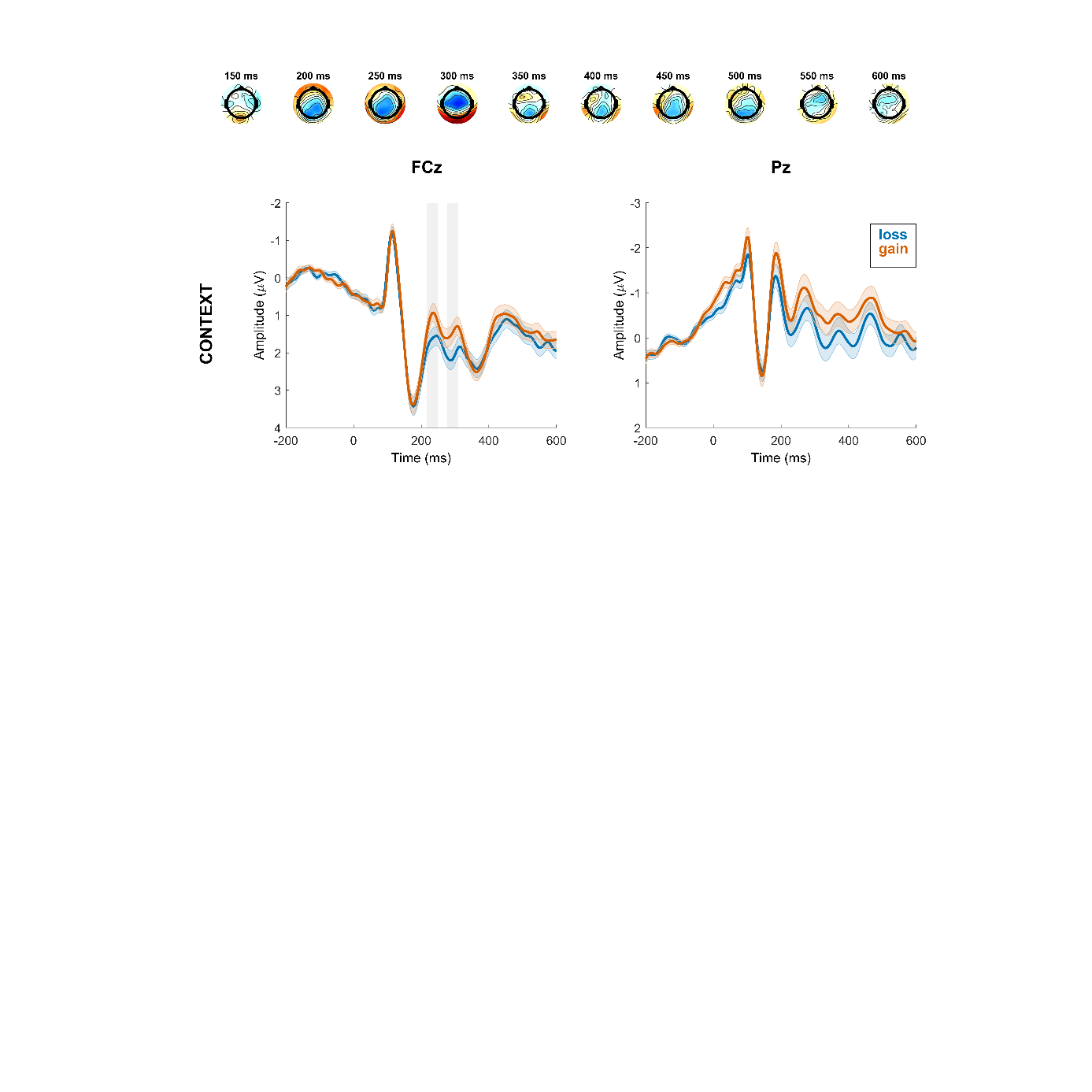 |
| --- |
| FIGURE 6 \| Time course of feedback-locked regression effects for regressor context (loss, gain). Grand average event-related potential (ERP) waveforms are depicted in the second row, feedback-locked for error trials. Shadows indicate the SEM. Gray shading behind the waveforms indicates significance. The first row represents topographical maps of the associations between EEG activity and the context regressor. |

**Cue-locked analysis**

The model for cue-locked EEG included a regressor coding the motivational context of the current trial (loss/gain), and the following regressors to account for factors of no interest: the accuracy (correct/error) of the last trial and the inter-trial interval (in ms). H_0_ was rejected for p = 5.0400e^-5^ (FCz) and p = 7.4600e^-5^ (Pz). The model revealed, amongst others, a significant covariation of context with cue-locked EEG activity, e.g. at the FCz in time windows corresponding to the cue-N1, with the loss context yielding higher amplitudes (124 to 170 ms), and the cue-N2, with the loss context yielding higher amplitudes (288 to 324 ms). The cue-N1 is associated with visual discriminative processing related to reward as well as attention [3,4]. The cue-N2 is associated with cognitive processing and proposed to present a template mismatch: participants possibly display a positivity bias or enhanced expectation towards reward cues, which in turn generates an enhanced amplitude following punishment cues because these deviate from the expectation [5-7]. Both therefore appear to be elevated due to higher attentional capture at this stage, possibly due to a positivity bias towards reward expectation.

The cue-P2 and cue-P3 did not differ in amplitude between contexts. Both have been associated with emotional and motivational salience as well as attention allocation [3-5,8,9]. Differential processing of reward and neutral cues with behavioral consequences and likely alterations in effort allocation have been reported in Flores, et al. [4]. As for the cue-P3, both reward as well as punishment cues have been reported to be associated with higher amplitudes, while there was no difference between reward and punishment cues [6,10]. As our task did not involve neutral incentive trials, no difference in cue-P2 and cue-P3 amplitudes may indicate comparable motivational salience for both incentive contexts, at least at the cue processing stage. See Figure 7 for a visualization of results.

| 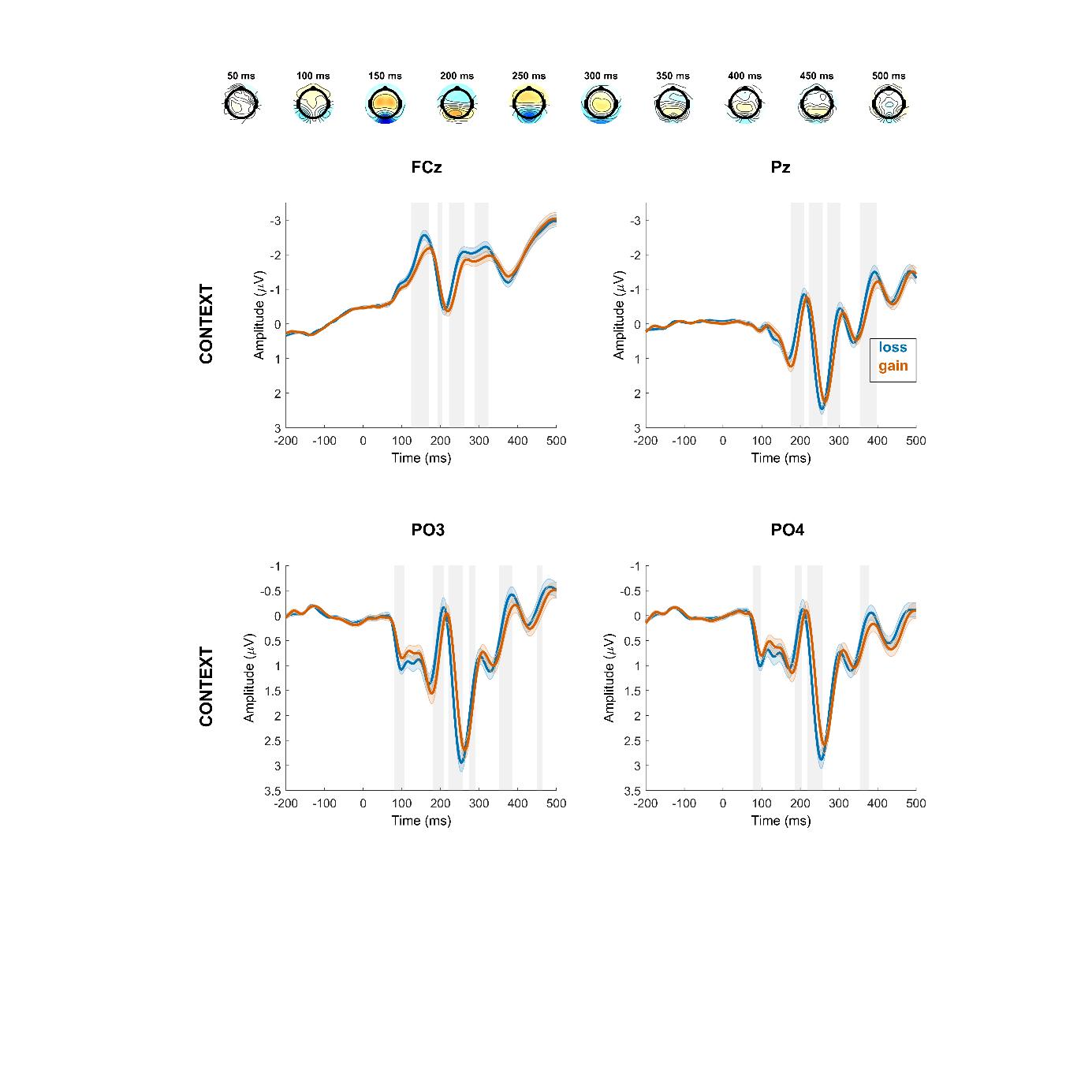 |
| --- |
| FIGURE 7 \| Time course of cue-locked regression effects for regressor context (loss, gain). Grand average event-related potential (ERP) waveforms are depicted in the second row, cue-locked for all trials. Shadows indicate the SEM. Gray shading behind the waveforms indicates significance. The first row represents topographical maps of the associations between EEG activity and the context regressor. |

References

1 Cohen, J. D., Botvinick, M. & Carter, C. S. Anterior cingulate and prefrontal cortex: who's in control? *Nature neuroscience* **3**, 421 (2000).

2 Benjamini, Y. & Yekutieli, D. The control of the false discovery rate in multiple testing under dependency. *Annals of statistics*, 1165-1188 (2001).

3 Doñamayor, N., Schoenfeld, M. A. & Münte, T. F. Magneto-and electroencephalographic manifestations of reward anticipation and delivery. *Neuroimage* **62**, 17-29 (2012).

4 Flores, A., Münte, T. F. & Donamayor, N. Event-related EEG responses to anticipation and delivery of monetary and social reward. *Biological psychology* **109**, 10-19 (2015).

5 Carlson, J. M. A systematic review of event‐related potentials as outcome measures of attention bias modification. *Psychophysiology* **58**, e13801 (2021).

6 Glazer, J. E., Kelley, N. J., Pornpattananangkul, N., Mittal, V. A. & Nusslock, R. Beyond the FRN: Broadening the time-course of EEG and ERP components implicated in reward processing. *International Journal of Psychophysiology* **132**, 184-202 (2018).

7 Folstein, J. R. & Van Petten, C. Influence of cognitive control and mismatch on the N2 component of the ERP: a review. *Psychophysiology* **45**, 152-170 (2008).

8 Yeung, N. & Sanfey, A. G. Independent coding of reward magnitude and valence in the human brain. *Journal of Neuroscience* **24**, 6258-6264 (2004).

9 Polich, J. & Kok, A. Cognitive and biological determinants of P300: an integrative review. *Biological psychology* **41**, 103-146 (1995).

10 Pfabigan, D. M. *et al.* P300 amplitude variation is related to ventral striatum BOLD response during gain and loss anticipation: an EEG and fMRI experiment. *NeuroImage* **96**, 12-21 (2014).
